# Cerebrovascular pressure reactivity and brain tissue oxygen monitoring provide complementary information regarding the lower and upper limits of cerebral blood flow control in traumatic brain injury: a CAnadian High Resolution-TBI (CAHR-TBI) cohort study

**DOI:** 10.1186/s40635-022-00482-3

**Published:** 2022-12-23

**Authors:** Alwyn Gomez, Mypinder Sekhon, Donald Griesdale, Logan Froese, Eleen Yang, Eric P. Thelin, Rahul Raj, Marcel Aries, Clare Gallagher, Francis Bernard, Andreas H. Kramer, Frederick A. Zeiler

**Affiliations:** 1 Department of Human Anatomy and Cell Science, Rady Faculty of Health Sciences, University of Manitoba, Winnipeg, Canada; 2 Present Address: Section of Neurosurgery, Department of Surgery, Rady Faculty of Health Sciences, University of Manitoba, Winnipeg, MB Canada; 3 Biomedical Engineering, Faculty of Engineering, University of Manitoba, Winnipeg, MB Canada; 4 Department of Anesthesiology, Pharmacology & Therapeutics, University of British Columbia, Vancouver, BC Canada; 5 Department of Neurology, Karolinska University Hospital, Stockholm, Sweden; 6 Department of Clinical Neuroscience, Karolinska Institutet, Stockholm, Sweden; 7 Department of Neurosurgery, University of Helsinki and Helsinki University Hospital, Helsinki, Finland; 8 Department of Intensive Care, Maastricht University Medical Center, Maastricht, The Netherlands; 9 Section of Neurosurgery, Department of Clinical Neurosciences, University of Calgary, Calgary, Canada; 10 Section of Critical Care, Department of Medicine, University of Montreal, Montreal, QC Canada; 11 Department of Critical Care Medicine, University of Calgary, Calgary, Canada; 12 Centre On Aging, University of Manitoba, Winnipeg, Canada; 13 Division of Anaesthesia, Department of Medicine, Addenbrooke’s Hospital, University of Cambridge, Cambridge, UK; 14 Department of Clinical Neurosciences, University of Calgary, Calgary, Canada; 15 Hotchkiss Brain Institute, University of Calgary, Calgary, Canada; 16 Present Address: Division of Critical Care, Department of Medicine, University of British Columbia, Vancouver, BC Canada; 17 School of Mental Health and Neurosciences, University Maastricht, Maastricht, The Netherlands

**Keywords:** Traumatic brain injury, Brain tissue oxygen tension, Cerebrovascular reactivity, Multi-modal monitoring, Cerebrovascular physiology

## Abstract

**Background:**

Brain tissue oxygen tension (PbtO_2_) and cerebrovascular pressure reactivity monitoring have emerged as potential modalities to individualize care in moderate and severe traumatic brain injury (TBI). The relationship between these modalities has had limited exploration. The aim of this study was to examine the relationship between PbtO_2_ and cerebral perfusion pressure (CPP) and how this relationship is modified by the state of cerebrovascular pressure reactivity.

**Methods:**

A retrospective multi-institution cohort study utilizing prospectively collected high-resolution physiologic data from the CAnadian High Resolution-TBI (CAHR-TBI) Research Collaborative database collected between 2011 and 2021 was performed. Included in the study were critically ill TBI patients with intracranial pressure (ICP), arterial blood pressure (ABP), and PbtO_2_ monitoring treated in any one of three CAHR-TBI affiliated adult intensive care units (ICU). The outcome of interest was how PbtO_2_ and CPP are related over a cohort of TBI patients and how this relationship is modified by the state of cerebrovascular reactivity, as determined using the pressure reactivity index (PRx).

**Results:**

A total of 77 patients met the study inclusion criteria with a total of 377,744 min of physiologic data available for the analysis. PbtO_2_ produced a triphasic curve when plotted against CPP like previous population-based plots of cerebral blood flow (CBF) versus CPP. The triphasic curve included a plateau region flanked by regions of relative ischemia (hypoxia) and hyperemia (hyperoxia). The plateau region shortened when cerebrovascular pressure reactivity was disrupted compared to when it was intact.

**Conclusions:**

In this exploratory analysis of a multi-institution high-resolution physiology TBI database, PbtO_2_ seems to have a triphasic relationship with CPP, over the entire cohort. The CPP range over which the plateau exists is modified by the state of cerebrovascular reactivity. This indicates that in critically ill TBI patients admitted to ICU, PbtO_2_ may be reflective of CBF.

**Supplementary Information:**

The online version contains supplementary material available at 10.1186/s40635-022-00482-3.

## Background

Despite traumatic brain injury (TBI) being a leading cause of death and disability worldwide [1], current guideline-based management, focused on intracranial pressure (ICP) and cerebral perfusion pressure (CPP), has resulted in limited recent improvements in outcome following moderate and severe TBI [2, 3]. Interest has shifted towards personalized medicine-based strategies that leverage contemporary multimodal monitoring techniques [4, 5]. Two methods that have come to the forefront are brain tissue oxygen tension (PbtO_2_) and ICP-based cerebrovascular pressure reactivity monitoring [6, 7].

PbtO_2_ monitoring requires the placement of a probe into viable brain tissue to measure oxygen tension [8]. A phase 2 randomized control trial (RCT) as well as observational studies have pointed towards improved outcomes when utilized in the TBI population [7, 9]. Consensus-based guidelines exist that outline the management of critically ill TBI patients with both ICP and PbtO_2_ monitoring and at least three large phase 3 RCTs are currently underway comparing PbtO_2_ augmented management to ICP and CPP based management alone in the setting of TBI [10–13].

Continuous cerebrovascular pressure reactivity monitoring uses the existing in situ ICP monitor [14]. While numerous methods exist, the pressure-reactivity index (PRx), which is the continuously computed correlation between slow-wave fluctuations in arterial blood pressure (ABP) and ICP, is the most studied and validated. PRx values range from − 1 to + 1 and cerebrovascular pressure reactivity is disrupted when there is a strong positive (> + 0.25) correlation between ICP and ABP [6]. Furthermore, a PRx above + 0.25 has been associated with worse neurologic outcomes following TBI [15, 16]. PRx is not directly modifiable, but the existence of a parabolic relationship between PRx and CPP in individual patients has led to the concept of personalized ‘optimal’ CPP management where individual PRx functions best [17, 18]. A full description of the computational methods is found elsewhere [17–21]. The output of this real-time analysis is a CPP at which PRx is at a minimum (CPPopt). The upper limit of reactivity (ULR) and lower limit of reactivity (LLR), are computed as the lower and upper limits of CPP values where PRx rises above + 0.25 [19, 22–25]. A management algorithm aiming to maintain CPP as close as possible to CPPopt has proven to be feasible and safe in a phase 2 RCT [21]. However, currently no Phase 3 data exist to support its efficacy in improving outcomes and no clinical guidelines are available for its use in TBI.

There are few studies examining the relationship between CPP, PRx and PbtO_2_ and none have leveraged contemporary data science techniques [26]. This work describes an exploratory multi-institutional retrospective cohort study that examines the interaction between these variables by leveraging prospectively collected high-resolution physiologic data from the CAnadian High Resolution-TBI (CAHR-TBI) Research Collaborative [27]. The primary aim of this study is to examine the relationship between PbtO_2_ and CPP in critically ill TBI patients. Secondary aims include: (1) examining how the relationship between PbtO_2_ and CPP is modified by the state of cerebrovascular pressure reactivity; (2) examining the relationship between PbtO_2_ and deviation from CPPopt, and (3) the independent prognostic utility of PbtO_2_ and CPPopt-based monitoring.

## Methods

### Study design

A retrospective multicenter cohort study utilizing a prospectively collected database of critically ill TBI patients was performed. The data originated from the CAnadian High Resolution-TBI (CAHR-TBI) Research Collaborative [27, 28]. Patients were admitted to one of three university-affiliated hospitals: Vancouver General Hospital (University of British Colombia), Foothills Medical Centre (University of Calgary), and Health Sciences Centre Winnipeg (University of Manitoba). Local research ethics approval at the University of Manitoba has been obtained for all aspects of this database (H2017:181 and H2017:188). Similarly, ethics approval was obtained for retrospective access to the database and for anonymous data transfer with each center for this project (H2020:118, H20-03,759 and REB20-0482).

### Patient population

The CAHR-TBI database includes TBI patients treated in an adult intensive care unit (ICU) with invasive ICP and ABP monitoring at one of the affiliated hospitals. All patients were cared for using contemporary management strategies based on Brain Trauma Foundation (BTF) guidelines [2]. However, CPP values greater than 70 mmHg were generally not therapeutically lowered. PbtO_2_ and cerebrovascular pressure reactivity was managed based on local practice norms and varied from aggressive management to purely observation. Granular patient-specific and center-specific differences in management were not well captured and were therefore unavailable for incorporation into this study. Typically, based on common practice patterns at participating institutions, similar vasopressor, sedative and hyperosmolar/hypertonic agents were utilized between all sites. Patients with mild TBI or without invasive ICP monitoring were excluded [27]. Patient data were entered into the database from 2011 to 2021.

Included in this study were all patients in the CAHR-TBI database that had concurrent invasive PbtO_2_ monitoring. Those without PbtO_2_ monitoring were excluded. Age, biologic sex, admission Glasgow Coma Score (GCS) motor score, admission pupil exam, and 6- to 12-month Glasgow Outcome Score (GOS) (based on individual site outcome assessment periods), were extracted when available. Given the exploratory nature of this study, sample size calculations were not possible and therefore not performed.

### High-resolution physiologic data collection

Three high-resolution physiologic data streams were utilized; ABP, ICP, and PbtO_2_. ABP was measured utilizing radial arterial lines. ICP was monitored using intra-parenchymal strain gauge probes (Codman ICP MicroSensor; Codman & Shurtlef Inc., Raynham, MA) placed in the frontal lobe or using external ventricular drains (Medtronic, Minneapolis, MN). PbtO_2_ was measured using intra-parenchymal brain tissue oxygenation probes (Licox Brain Tissue Oxygen Monitoring System; Integra LifeSciences Corp., Plainsboro, New Jersey) placed in viable frontal lobe tissue.

Data streams were recorded in digital high-frequency time series (≥ 100 Hz for ABP and ICP, 1 Hz for PbtO_2_) using analogue-to-digital signal converters (Data Translations, DT9804 or DT9826) where applicable. This digitized data was linked and stored in time series using Intensive Care Monitoring (ICM +) software (Cambridge Enterprise Ltd, Cambridge, UK).

### Physiologic data cleaning and processing

For all high-resolution physiologic data, artifact clearing was performed utilizing manual removal by a qualified clinician utilizing ICM + software. All data were cleaned without knowledge of patient demographic or outcome information.

For each patient dataset, ABP and ICP waveforms were decimated using a 10-s moving average filter to eliminate higher frequency oscillations in these signals using ICM + functions. The resulting signals were used to derive a minute-by-minute updating value of PRx using a continuously updating Pearson correlation between ABP and ICP [17]. CPP was also derived as the difference between ABP and ICP. CPPopt, ULR, and LLR were all computed as continuously updated time series variables, where possible, utilizing a weighted multi-window technique examining the parabolic relationship between CPP and PRx as described recently [19, 21, 23–25]. A PRx value of + 0.25 was utilized as the threshold to determine both the ULR and LLR based on previous literature [16]. No attempt was made to interpolate missing data as entirely continuous data streams were not required for the subsequent analysis. Finally, all recorded and derived physiologic data (ABP, ICP, PbtO_2_, CPP, PRx, CPPopt, ULR and LLR) were exported as minute-by-minute comma-separated value (CSV) files for use in data analysis.

### Physiologic data analysis and statistical methods

The data analysis was performed using R statistical software (R Foundation for Statistical Computing, Vienna, Austria). The data were isolated for values of PbtO_2_ greater than 0 mmHg, ABP greater than 20 mmHg, and CPP values between 20 and 150 mmHg as data outside of these parameters were likely artifactual. Next, a delta-CPPopt (ΔCPPopt) was calculated as the difference between CPP and CPPopt for every available datapoint. Finally, the following parameters were calculated for each subject in the study:

1. Percent time with CPP less than 60 mmHg and percent time with CPP greater than 70 mmHg as these are defined targets in BTF guidelines [2].
2. Percent time with PbtO_2_ less than 20 mmHg as this is a defined threshold in the literature [9–12].
3. Percent time with CPP less than the computed LLR and percent time with CPP above the computed ULR.

Plots were created using a generalized additive model smoothing function. This included visualization of the relationship between PbtO_2_ and CPP as well as the relationship between PbtO_2_ and ΔCPPopt over the entire cohort to identify global relationships. The effect of cerebrovascular pressure reactivity function, defined with a PRx threshold of 0.25, was also examined as a modifier to these relationships.

Finally, multivariable logistic regression modeling was performed to examine the utility of various parameters and exposures for predicting dichotomized 6- to 12-month functional outcomes (Favorable: GOS 4–5 vs Unfavorable: GOS 1–3 and Alive vs Dead). This was performed to gain insight into the possible impact these exposures may have on clinical outcomes following TBI. Models all included the International Mission for Prognosis and Analysis of Clinical Trials in TBI (TBI-IMPACT) variables of age, admission GCS motor score, admission pupil exam, and Marshall CT classification [29]. In total, six models were examined:

1. TBI-IMPACT.
2. TBI-IMPACT + Percent time with CPP less than 60 mmHg + Percent time with CPP greater than 70 mmHg.
3. TBI-IMPACT + Percent time with CPP less than 60 mmHg + Percent time with CPP greater than 70 mmHg + Percent time with PbtO_2_ less than 20 mmHg.
4. TBI-IMPACT + Percent time with CPP less than the computed LLR.
5. TBI-IMPACT + Percent time with CPP above the computed ULR.
6. TBI-IMPACT + Percent time with CPP less than the computed LLR + Percent time with CPP above the computed ULR.

Model quality was assessed utilizing area under the curve-receiver operator characteristic (AUC-ROC) analysis. Additionally, the quality of each model was further examined through Akaike Information Criteria (AIC) and Nagelkerke pseudo-R^2^ analysis. The alpha was set at 0.05 and due to the exploratory nature of the analysis, correction for multiple comparisons was not performed.

## Results

### Cohort demographics

In total, 77 patients from the CAHR-TBI database met the inclusion criteria for this study (57 from the University of British Colombia, 12 from the University of Calgary, and 8 from the University of Manitoba). Demographic data for the cohort are summarized in Table 1.

**Table 1:** Patient demographics for the cohort

Demographic parameter	Median or number of subjects
Age (IQR)	41 (25–57)
Gender	
Male subjects (%)	62 (80.5)
Female subjects (%)	14 (18.2)
N/A (%)	1 (1.3)
Admission GCS	
Eye (IQR)	1 (1–1)
Verbal (IQR)	1 (1–1)
Motor (IQR)	1 (1–4)
Total (IQR)	3 (3–6)
Admission pupils	
Bilaterally reactive (%)	62 (80.5)
Unilaterally reactive (%)	6 (7.8)
Bilaterally unreactive (%)	8 (10.4)
N/A (%)	1 (1.3)
Marshall CT classification	
I (%)	0 (0)
II (%)	29 (37.7)
III (%)	19 (24.7)
IV (%)	6 (7.8)
V (%)	4 (5.2)
VI (%)	2 (2.6)
N/A, n (%)	17 (22.1)
Follow-up GOS	
1 (%)	17 (22.1)
2 (%)	1 (1.3)
3 (%)	11 (14.3)
4 (%)	18 (23.4)
5 (%)	13 (16.9)
N/A, n (%)	17 (22.1)
Percent time PbtO_2_ less than 20 mmHg (IQR)	12.3 (4.0–28.9)
Percent time CPP less than 60 mmHg (IQR)	2.1 (0.5–4.3)
Percent time CPP greater than 70 mmHg (IQR)	86.6 (74.0–95.7)

*GCS* *Glasgow Coma Scale, CT* *computerized tomography, GOS* *Glasgow Outcome Scale, IQR* *interquartile range, N/A* *not available*

### Physiologic data

Following artifact clearing and data filtering, a total of 377,744 min of unique physiologic data recordings were available for analysis in this cohort with a median of 4882 min (IQR: 3219–7884 min) of physiologic data for each patient. The yield of the CPPopt calculations were acceptable with a median percent yield of 70% (IQR: 55–81%) for each patient [19, 21].

### Qualitative visual analysis

**Fig. 1:**
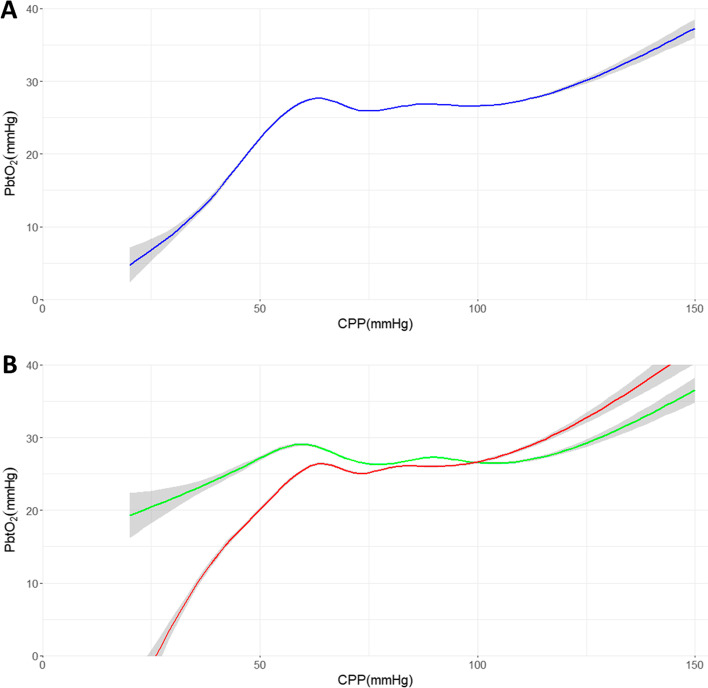
The relationship of brain tissue oxygenation (PbtO_2_) versus cerebral perfusion pressure (CPP) is seen in (**A**). In **B**, this relationship is examined when cerebrovascular pressure reactivity is intact (green) as compared to when it is disrupted (red). Note that 95% confidence intervals for all tracings are shown in grey

The relationship between PbtO_2_ and CPP over the cohort can be seen in Fig. 1A. The plot resembles the classical, population-based, triphasic Lassen curve with a plateau between CPP values of approximately 60 mmHg to 110 mmHg [30]. Below this plateau region, PbtO_2_ sharply falls and above it PbtO_2_ sharply rises. Interestingly the plateau region has associated PbtO_2_ values between 25 and 30 mmHg which is notably higher than the current guideline-based treatment threshold of 20 mmHg [7, 9–12]. Unlike the classic population-based Lassen curve there are two distinct humps that occur within the plateau region.

In Fig. 1B, the recorded physiologic data are divided based on if cerebrovascular pressure reactivity was intact (green) or disrupted (red) using a PRx threshold of + 0.25. In both tracings a variation of the classic triphasic, population-based, Lassen curve is seen [30], however, the plateau region is substantially expanded when PRx is less than + 0.25 than when it is greater than + 0.25. Again, the plateau region is associated with PbtO_2_ values between 25 and 30 mmHg. When cerebrovascular pressure reactivity is intact, two distinct humps occur within the plateau region. When cerebrovascular pressure reactivity is disrupted the hump at higher CPP values is interrupted, shortening the plateau region. Individual patient plots, found in Additional file 1: Appendix SA, did not consistently demonstrate this pattern.

**Fig. 2:**
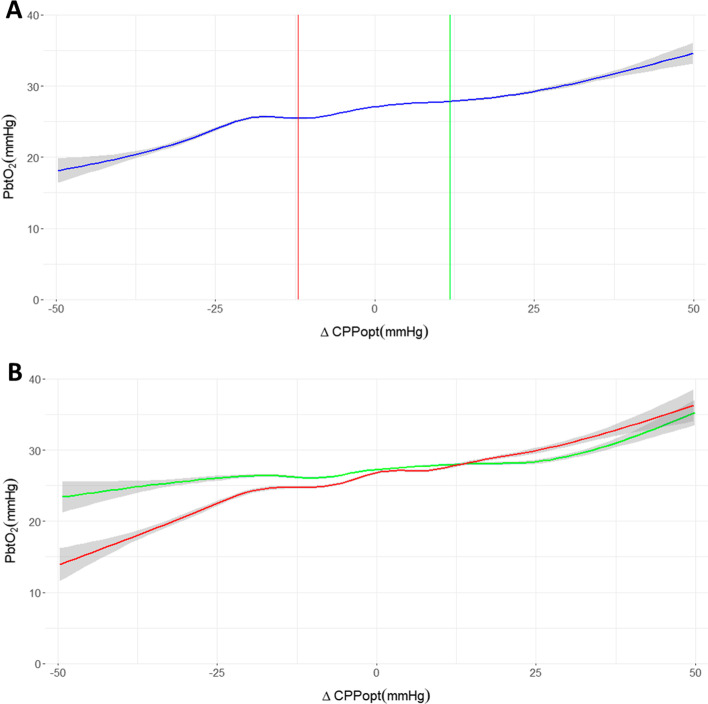
The relationship between brain tissue oxygenation (PbtO_2_) and difference from optimal cerebral perfusion pressure (ΔCPPopt) is seen in **A**. The median ΔCPPopt value associated with the lower limit of reactivity (red) and upper limit of reactivity (green) are also plotted. In **B**, this relationship is examined when cerebrovascular pressure reactivity is intact (green) as compared to when it is disrupted (red). Note that 95% confidence intervals for all tracings are shown in grey

The relationship between PbtO_2_ and ΔCPPopt over the cohort is presented in Fig. 2A. Additionally, the median ΔCPPopt associated with the LLR and ULR in the cohort are shown in red and green, respectively. Once again, a triphasic curve is observed with a plateau region containing two humps. The CPPopt, and the median ΔCPPopt associated with the LLR and ULR appear to be primarily centered around the hump at higher CPP values. In Fig. 2B, this relationship is examined when cerebrovascular pressure reactivity is intact (green) versus when it is disrupted (red). As with Fig. 1b while both curves contain a plateau region, that region spans a much larger range when cerebrovascular pressure reactivity is intact. Again, the plateau is found to be associated with a PbtO_2_ between 25 and 30 mmHg. Individual patient plots, found in Additional file 1: Appendix SB, did not consistently demonstrate this pattern.

### Functional outcome models

The results of the multivariable logistic regression, AUC, AIC, and Nagelkerke pseudo-R^2^ analysis can be seen in Table 2. Multivariable logistic regression showed that none of the individual factors were statistically significant. There was no clear trend for models incorporating percent time with PbtO_2_ less than 20 mmHg, percent time below the LLR, and/or percent time above the ULR to perform better than those that were based on TBI-IMPACT variables and constant CPP parameters alone. The superiority of the PbtO_2_-based model over CPPopt-based models, or vice versa, was also equivocal.

**Table 2:** Performance of various multivariable logistic regression models to predict 6-month post-injury outcomes

Model	Favorable vs unfavorable outcome			Alive vs dead		
	AUC (95% CI)	AIC	Nagelkerke R^2^	AUC (95% CI)	AIC	Nagelkerke R^2^
TBI-IMPACT	0.86 (0.76–0.96)	66.00	0.51	0.78 (0.66–0.90)	68.37	0.27
TBI-IMPACT + % Time CPP < 60 mmHg + % Time CPP > 70 mmHg	0.86 (0.76–0.96)	68.81	0.53	0.81 (0.69–0.93)	69.44	0.33
TBI-IMPACT + % Time CPP < 60 mmHg + % Time CPP > 70 mmHg + % Time PbtO_2_ < 20 mmHg	0.87 (0.77–0.96)	69.83	0.54	0.81 (0.70–0.93)	71.15	0.34
TBI-IMPACT + % Time CPP < LLR	0.86 (0.76–0.96)	65.81	0.53	0.80 (0.68–0.93)	65.85	0.32
TBI-IMPACT + % Time CPP > ULR	0.86 (0.77–0.96)	66.66	0.52	0.81 (0.69–0.92)	67.40	0.28
TBI-IMPACT + % Time CPP < LLR + % Time CPP > ULR	0.87 (0.77–0.96)	67.77	0.53	0.80 (0.68–0.93)	67.48	0.33

The TBI-IMPACT model included age, admission GCS, admission pupil exam, and Marshall score

*AIC* Akaike Information Criterion, *AUC* Area under the curve, *CPP* cerebral perfusion pressure, *GCS* Glasgow Coma Scale, *LLR* lower limit of reactivity, *PbtO*_*2*_ brain tissue oxygenation, *ULR* upper limit of reactivity

## Discussion

### Qualitative visual analysis

Through visualizing the relationship between PbtO_2_ and CPP or ΔCPPopt several interesting observations can be made. The first is that PbtO_2_, like cerebral blood flow (CBF), produces a triphasic curve when plotted against CPP or ΔCPPopt with a plateau region flanked by regions of relative ischemia (hypoxia) and hyperemia (hyperoxia). This plateau in PbtO_2_ over a range of CPP or ΔCPPopt values supports work previously conducted by Jaeger and colleagues in a smaller single-institution cohort [26].

The plateau region redemonstrates features recently identified by Klein et al. in a model of hypo- and hypertension in pigs with healthy brains [31]. In that study, which involved the intermittent measurement of CBF and pial vessel diameter through direct florescence microscopy by cranial window, the plateau region was composed of two distinct regions. Klein et al.’s study showed that at lower CPP values, within the plateau, the stability of CBF was attributable to the constriction of both small (< 70 µm) and large arterioles (> 70 µm), producing an initial hump in the plot of CBF versus CPP. At higher CPP values, within the plateau, stability in CBF was attributable to the constriction of larger arterioles while smaller arterioles were passively dilated, producing a second hump in the plateau region [31]. In the present study, these features are seen in the plateau regions of all cohort plots. In this cohort of critically ill TBI patients admitted to ICU, where oxygenation and hemoglobin concentrations are relatively stable, it is conceivable that PbtO_2_ primarily reflects changes in CBF attributable to changes in CPP. This may represent the first ever in human evidence of this biphasic plateau region, potentially produced by the action of small and large arterioles distinctly.

The plateau is truncated when cerebrovascular pressure reactivity is disrupted. This reinforces the notion that PRx is a valid measure of cerebral autoregulation in the traumatically injured brain. These results also indicate that PRx and cerebrovascular pressure reactivity PRx-based ΔCPPopt, LLR and ULR seem primarily sensitive to reactivity in larger arterioles. This can be seen in Fig. 1B where the morphology of the plateau hump associated with the isolated action of large arterioles is notably different when cerebrovascular pressure reactivity is disrupted. Additionally, in Fig. 2A the CPPopt (i.e., where ΔCPPopt equals zero) and LLR and ULR seem to be centered around the plateau region attributable to the action of large arterioles. This is not surprising as PRx relies on ICP as a surrogate for cerebral blood volume, which is impacted substantially more by the dilation and constriction of large arterioles than small ones [14].

Finally, the plateau region is associated with PbtO_2_ values of 25 mmHg to 30 mmHg. Current guideline-based management of PbtO_2_ in critically ill TBI patients primarily targets a PbtO_2_ greater than 20 mmHg [9–12]. The findings of this study point to a physiologically stable region of PbtO_2_ higher than currently targeted. Over this cohort, when CPP was found to be between the LLR and ULR, PbtO_2_ values were largely found to be within this range indicating that CPPopt-based management may be a suitable alternative when PbtO_2_ monitoring is unavailable.

### Functional outcome models

Neither PbtO_2_-based or CPPopt-based logistic regression models were found to be statistically superior to the TBI-IMPACT model at predicting functional outcomes. The significant proportion of missing outcome data in this small cohort means that this study was underpowered to identify a statistical difference. No clear trend was identified when comparing models that incorporated PbtO_2_ and those that incorporated CPPopt-based parameters. However, it should be noted that since PbtO_2_ was actively managed in some institutions, the prognostic effect of PbtO_2_ might have been artificially diminished.

### Limitations

While this study has significant strengths, such as its use of high-resolution physiologic data collected over multiple geographically distinct regions, there are notable limitations. As an observational study, confounding unmeasured physiologic parameters are possible such as heterogeneity in management strategies. Secondly, the validity of the generated functional outcome models is limited by missing data. Additionally, the yield of CPPopt, LLR, and ULR was lower than that found in previous studies, however this may just represent the real-world performance of these methods [19]. Third, given that the partial pressure of oxygen in the arterial blood (PaO_2_), oxygen saturation (SpO_2_), hemoglobin values, and various clinical management details were not available for all patients, changes in PbtO_2_ with changes in CPP cannot wholly be attributable to changes in CBF. This is particularly significant given that patient management likely varied between patients and between participating institutions and has not been able to be accounted for in the presented analysis. Finally, data were examined over the entire cohort, and so the validity of these relationships in individuals needs to be further examined prospectively.

### Future directions

This study does provide meaningful guidance for future research. Given that PbtO_2_ seems to have a similar relationship to CPP as CBF in the critically ill TBI, it may serve as an appropriate surrogate for microvascular CBF in this population. This will need to be validated in prospective studies in both humans and large animal models that incorporate PaO_2_, SpO_2_, and hemoglobin measurements.

The observation that a physiologic region of stability exists with PbtO_2_ values between 25 and 30 mmHg also warrants further exploration. While RCTs are currently underway to determine the utility of PbtO_2_ monitoring, current consensus-based guidelines and study protocols have centered around a treatment threshold of 20 mmHg [9–12]. The findings of this study indicate that more work is needed to identify an optimal target range for PbtO_2_.

The cerebrovascular pressure reactivity findings also need to be explored. Large animal models may help better understand the degree to which PRx favors the vascular reactivity of large arterioles over small ones. Additionally, the role of CPPopt in prognostication and personalized targeted therapy must also be explored as in this study, when CPP is near CPPopt, values of PbtO_2_ are reassuring. One major hurdle that will need to be overcome is the suboptimal yield of CPPopt derivation as well as its related parameters. While the multi-window technique utilized in this study represents an ongoing evolution of these algorithms, further improvements may aid its adoption as a tool to guide clinical management.

## Conclusion

In this exploratory analysis of a multi-institution high-resolution physiology TBI database, PbtO_2_ seems to have a triphasic relationship with CPP and ΔCPPopt that resembles a traditional, population-based, Lassen curve over the entire cohort [30]. The morphology of the plateau region is similar to that found in a recent large animal study evaluating the relationship between intermittent CBF and CPP, which may provide insights into the physiologic mechanisms that are involved [31]. In critically ill TBI patients admitted to ICU, PbtO_2_ may serve as a surrogate for CBF. The plateau region is associated with PbtO_2_ values of 25 mmHg to 30 mmHg which may help inform future PbtO_2_-based management. Notably, the CPP range over which the plateau exists is modified by the state of cerebrovascular pressure reactivity. Finally, there is some early evidence that CPPopt has potential as an alternative when PbtO_2_ monitoring is not available.

## Supplementary Information

**Additional file 1: Appendix SA.** Plots of PbtO_2_ versus CPP for individual patients. **Appendix SB.** Plots of PbtO_2_ versus ΔCPPopt for individual patients.

## Data Availability

The datasets used and analyzed during the current study are available from the corresponding author on reasonable request.

## Abbreviations

ABP: Arterial blood pressure
AIC: Akaike information criterion
AUC-ROC: Area under the curve of the receiver operator characteristic
BTF: Brain trauma foundation
CAHR-TBI: CAnadian High Resolution-TBI Research Collaborative
CBF: Cerebral blood flow
CPP: Cerebral perfusion pressure
CPPopt: Optimal cerebral perfusion pressure determined by PRx
CSV: Comma-separated value
CT: Computerized tomography
ΔCPPopt: Difference between CPP and CPPopt
GCS: Glasgow Coma Score
GOS: Glasgow Outcome Score
ICM +: Intensive care monitoring software
ICP: Intracranial pressure
ICU: Intensive care units
IQR: Interquartile range
LLR: Lower limit of reactivity
N/A: Not available
PaO_2_: Partial pressure of oxygen in the arterial blood
PbtO_2_: Brain tissue oxygen tension
PRx: Pressure reactivity index
RCT: Randomized control trial
SpO_2_: Oxygen saturation
TBI: Traumatic brain injury
TBI-IMPACT: International Mission for Prognosis and Analysis of Clinical Trials in TBI
ULR: Upper limit of reactivity
