# Supplementary material for: Cerebrovascular pressure reactivity and brain tissue oxygen monitoring provide complementary information regarding the lower and upper limits of cerebral blood flow control in traumatic brain injury: a CAnadian High Resolution-TBI (CAHR-TBI) cohort study
Source: Intensive Care Med Exp. 2022 Dec 23;10:54. doi: 10.1186/s40635-022-00482-3 (PMC9780411; doi:10.1186/s40635-022-00482-3)

**Appendix A – Plots of PbtO_2_ versus CPP for individual patients**


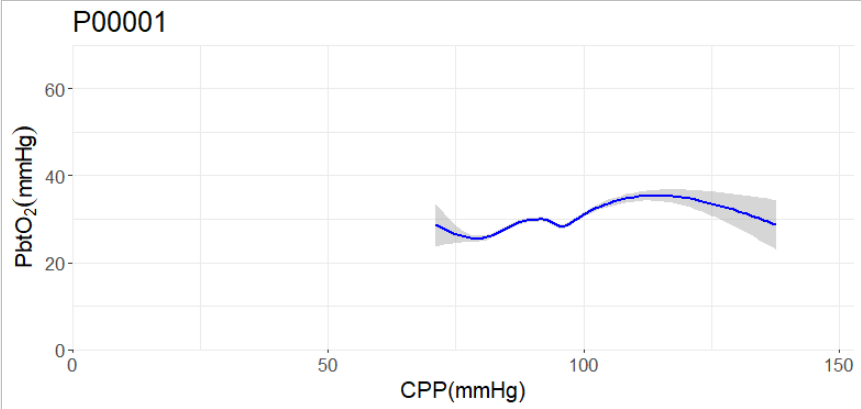


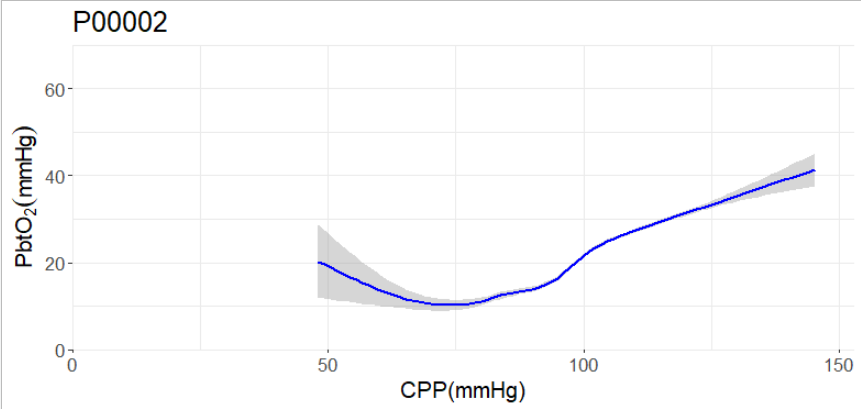


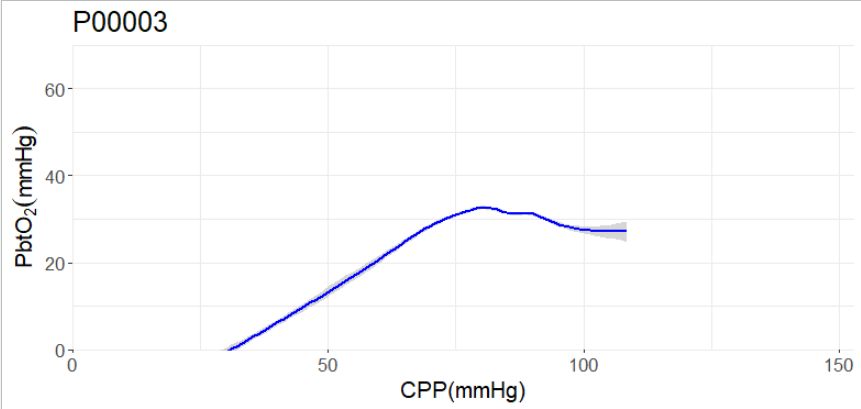


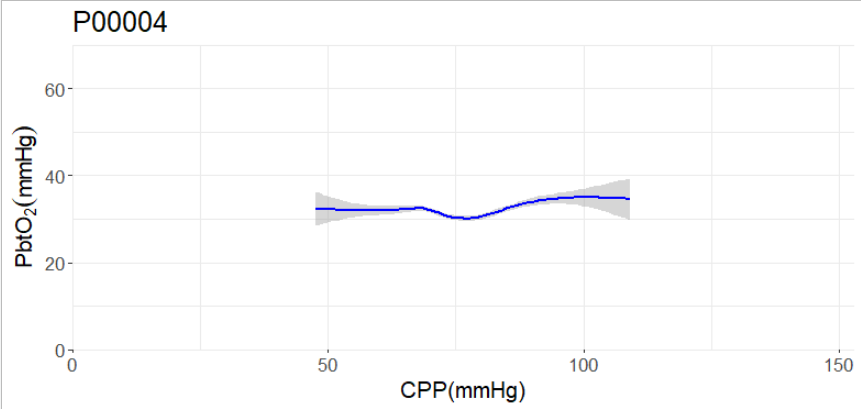


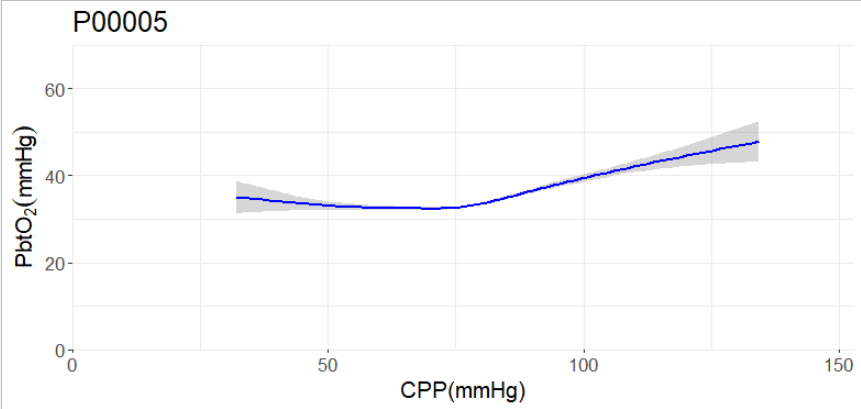


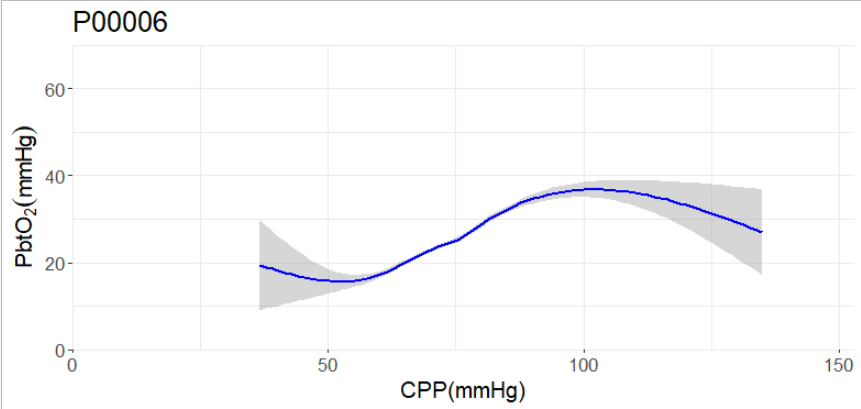


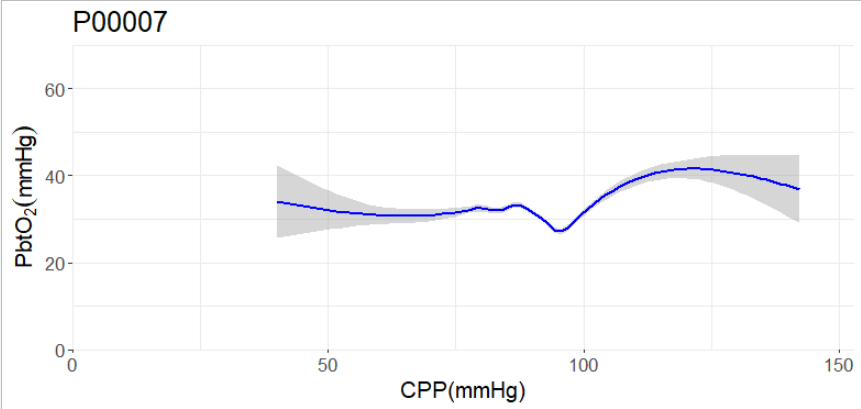


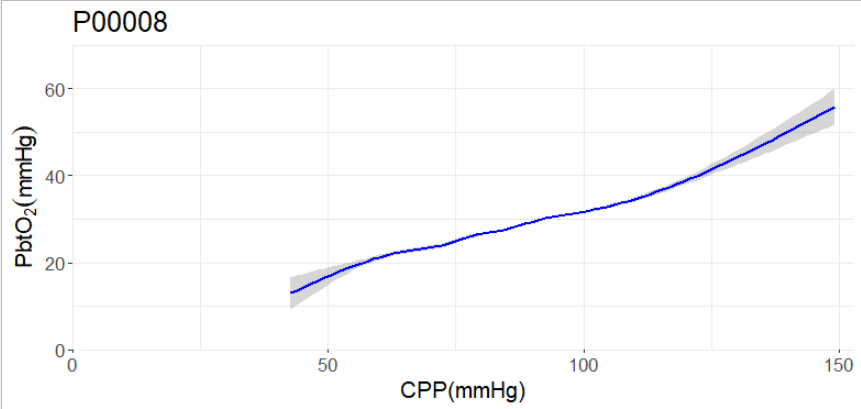


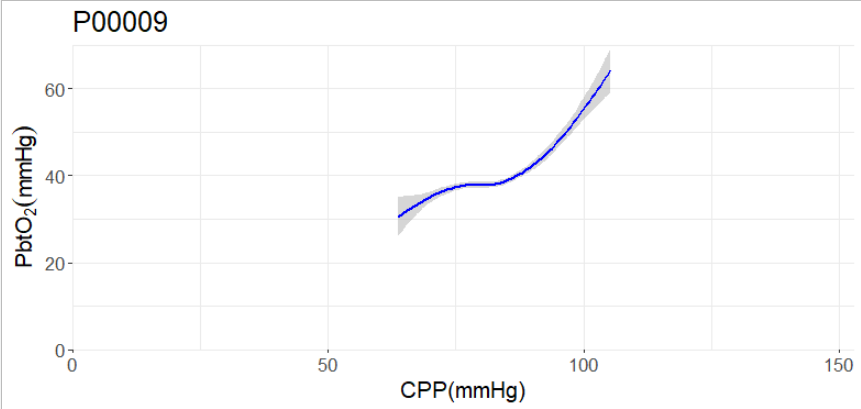


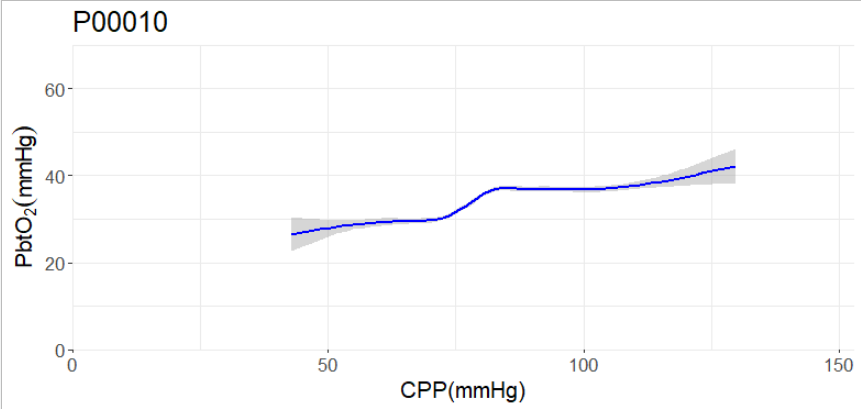


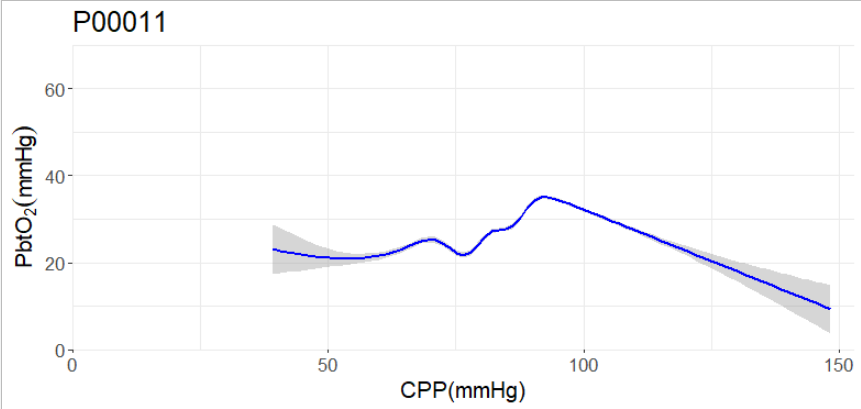


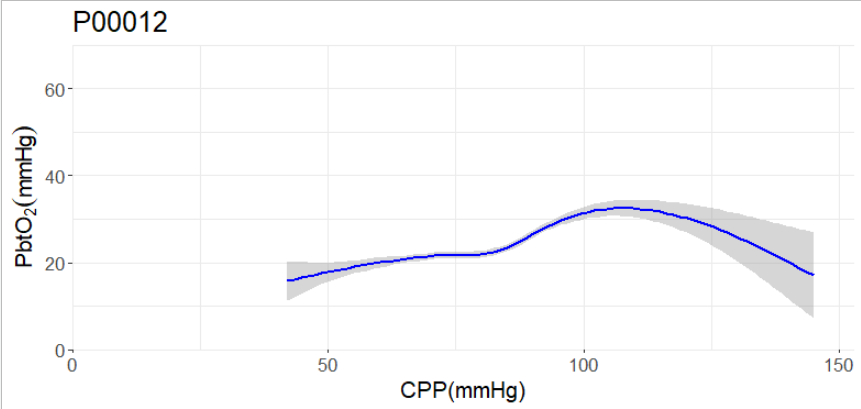


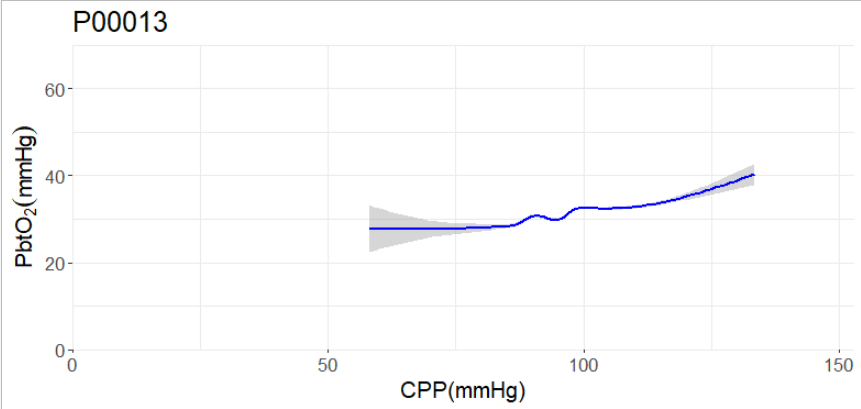


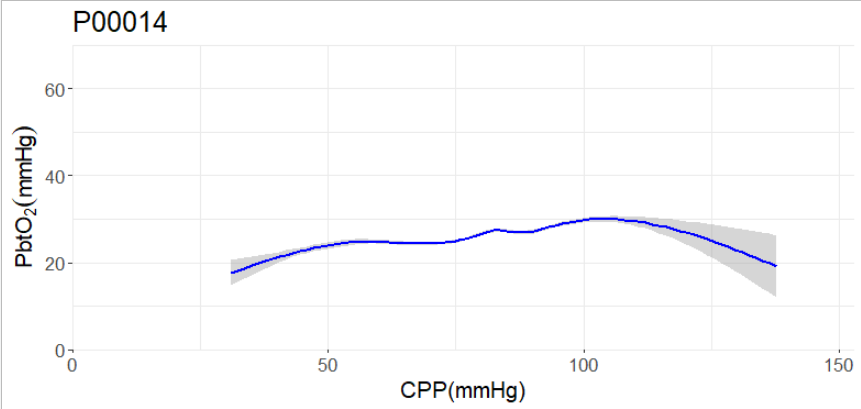


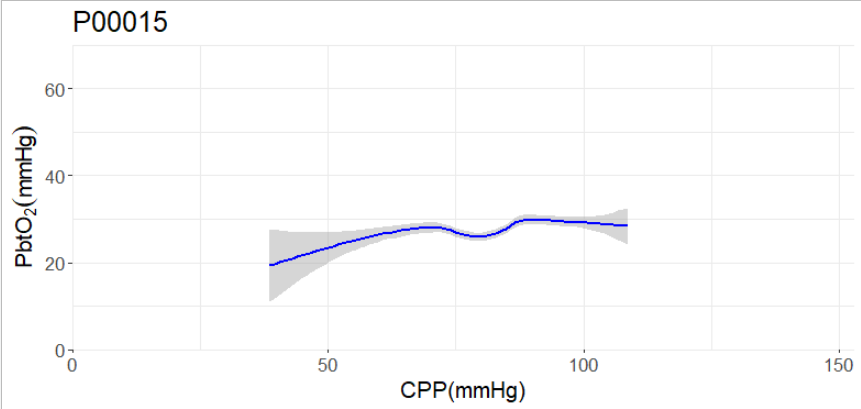


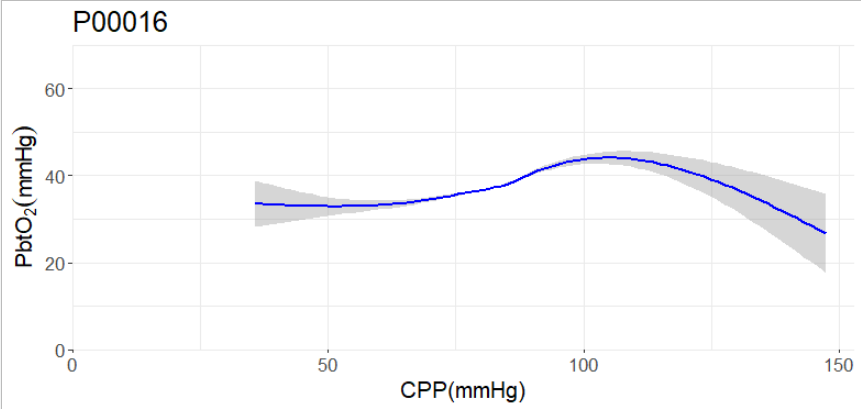


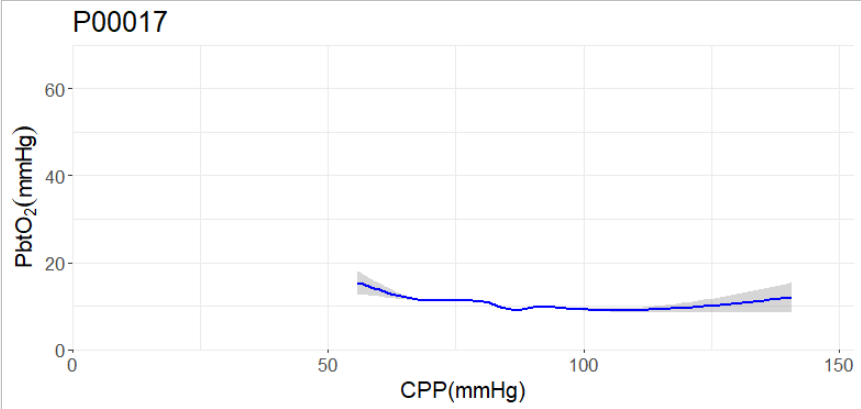


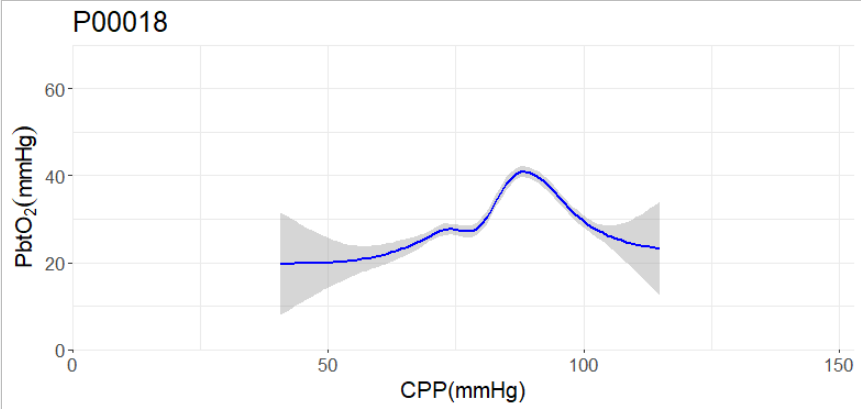


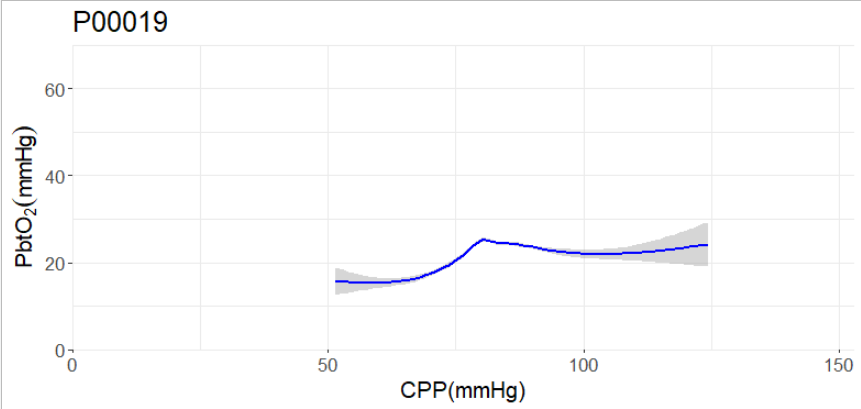


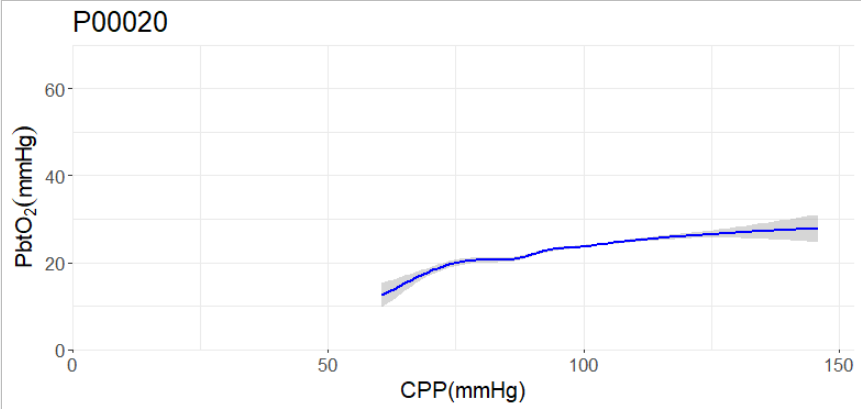


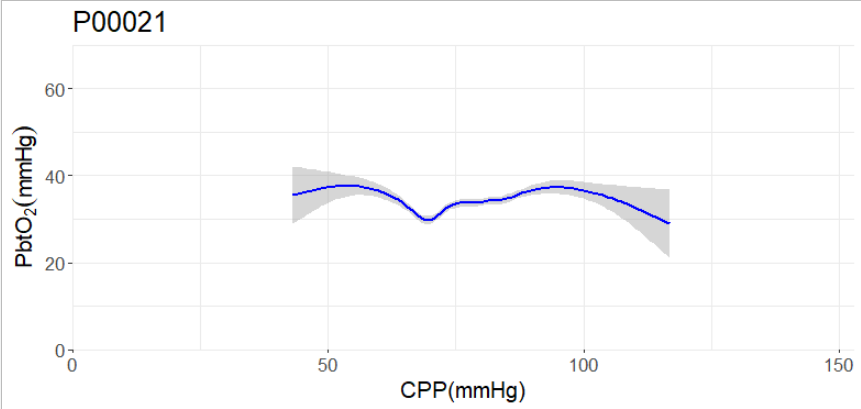


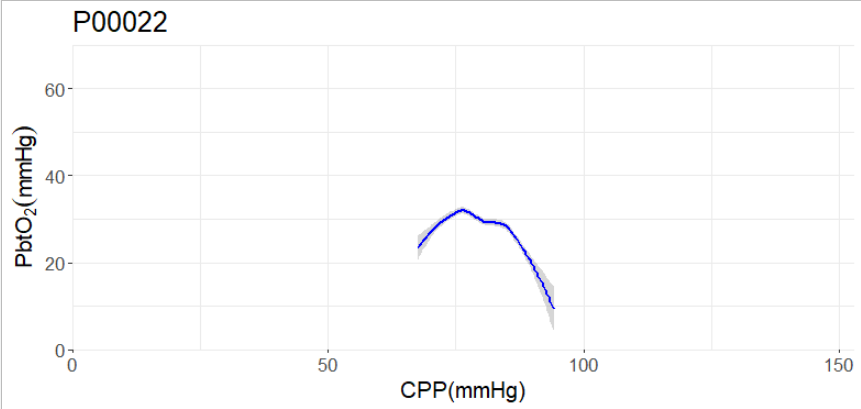


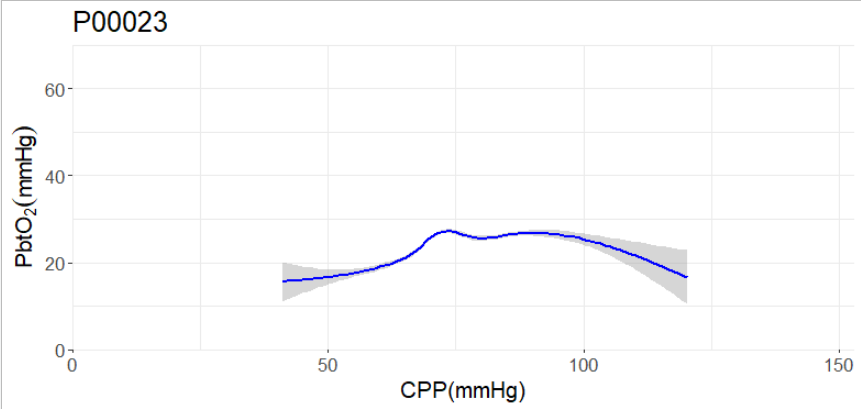


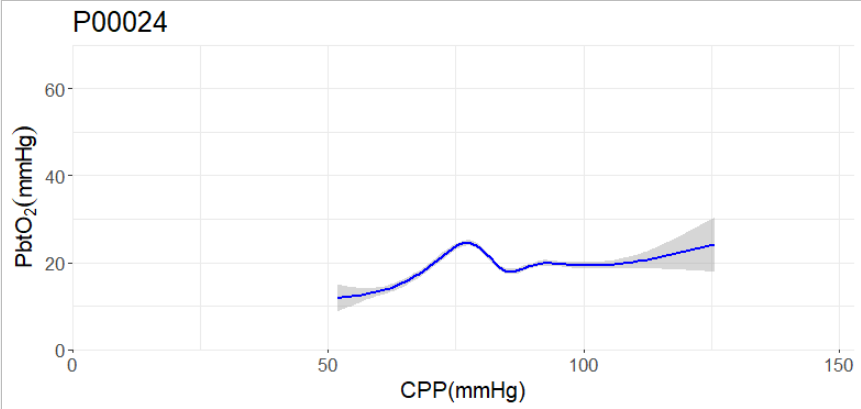


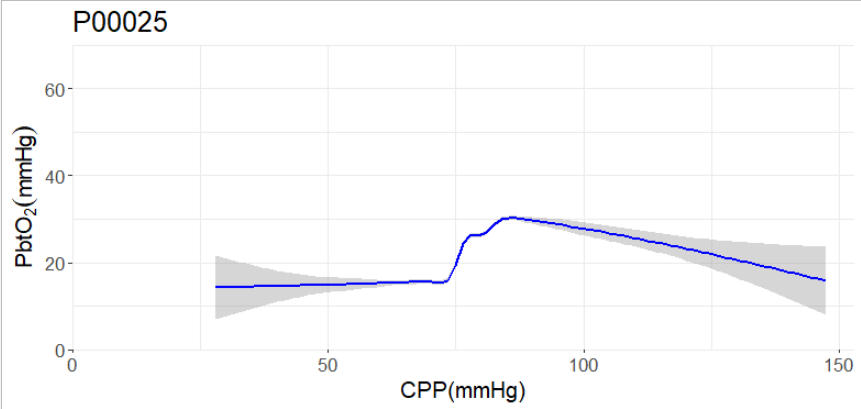


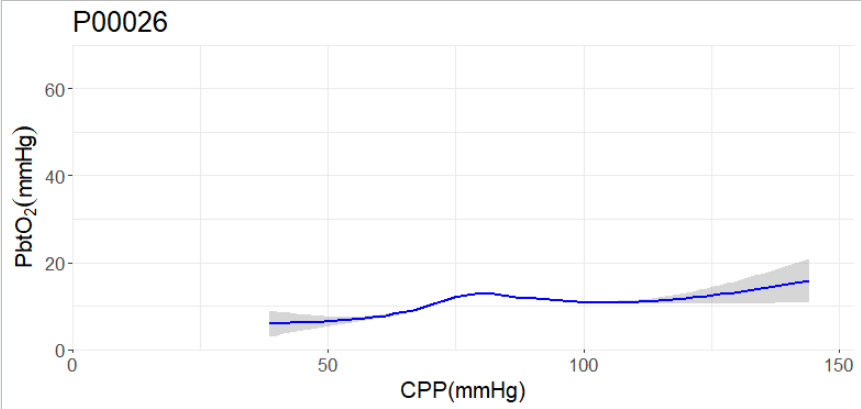


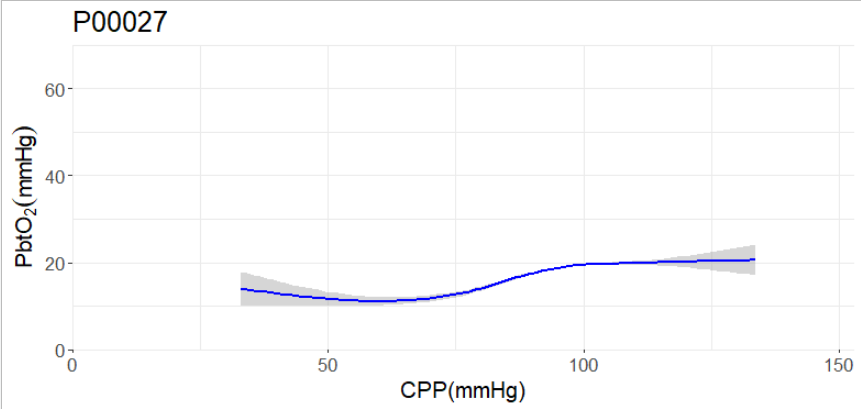


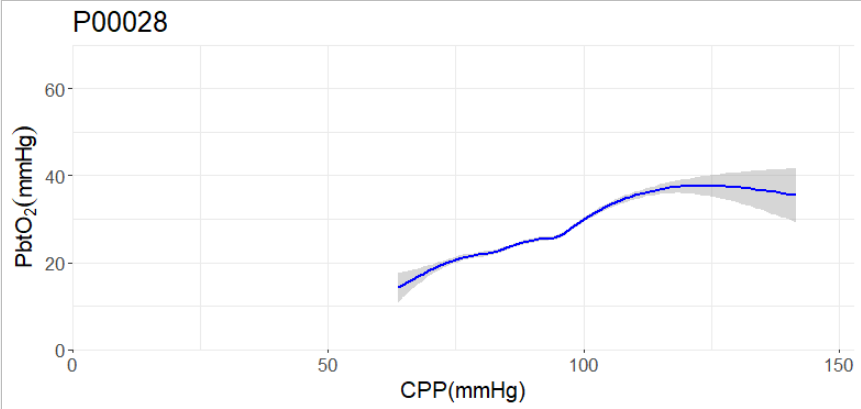


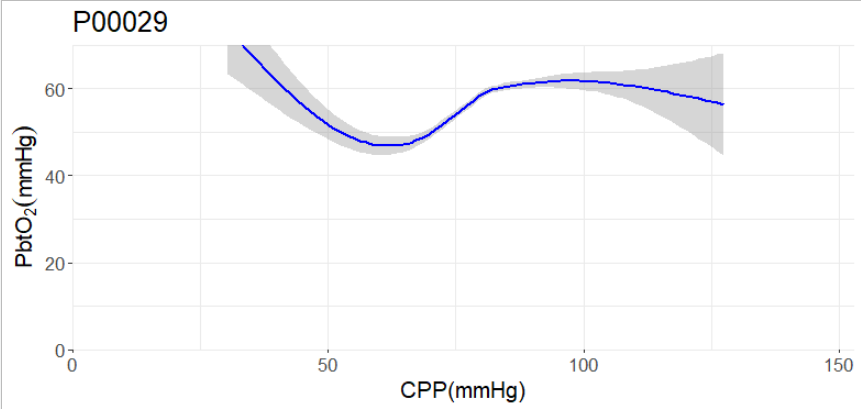


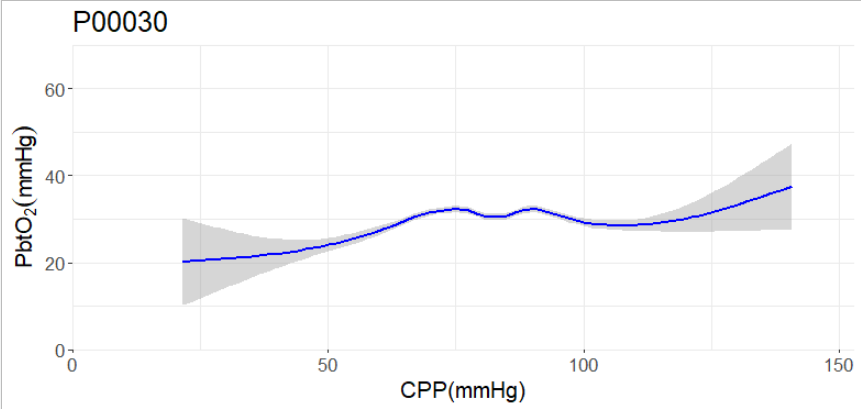


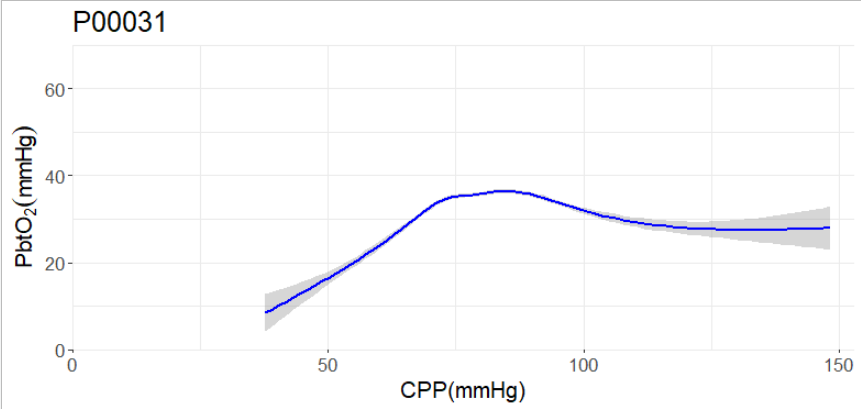


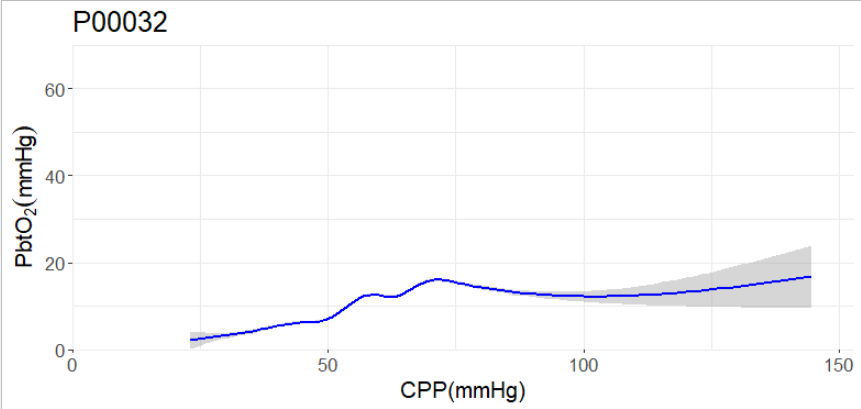


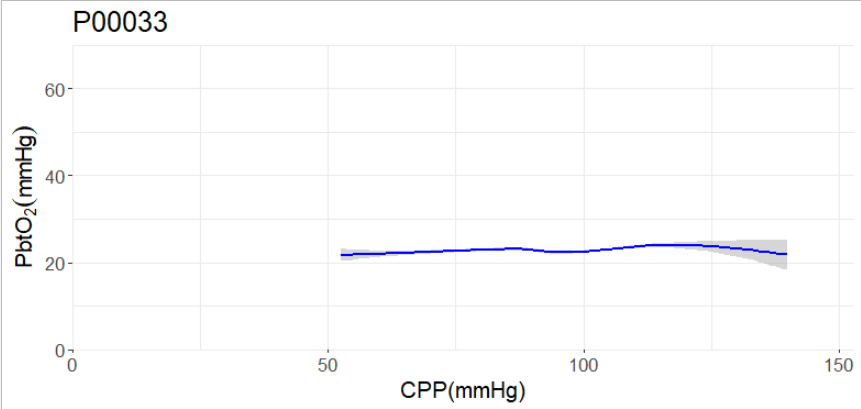


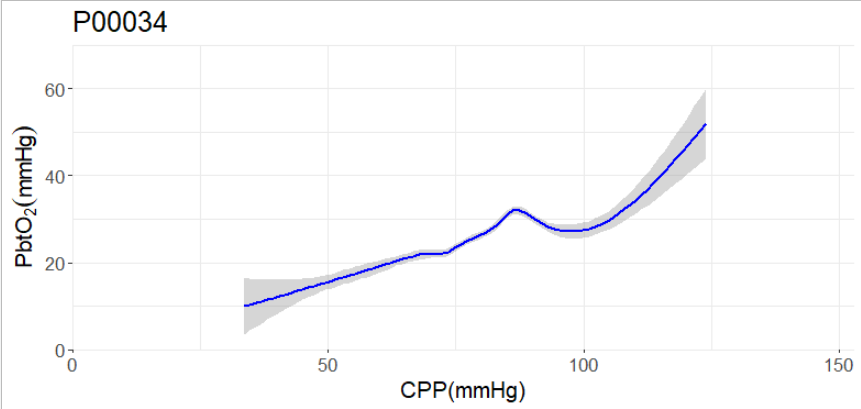


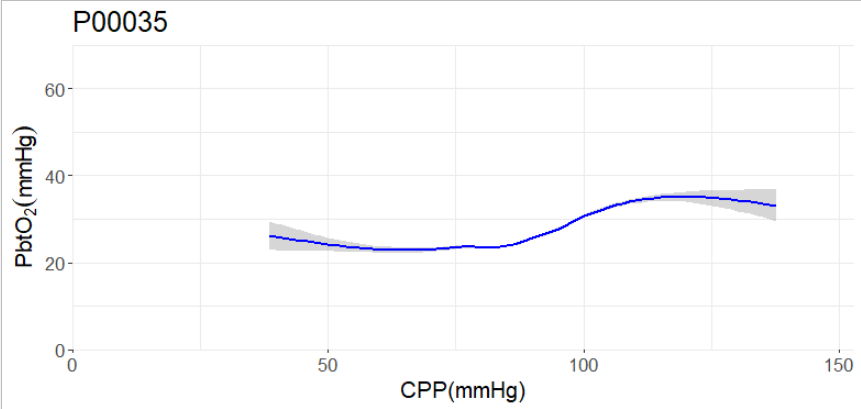


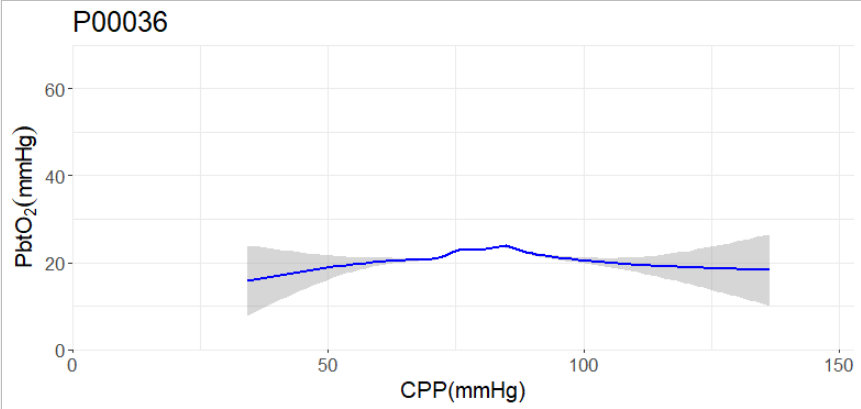


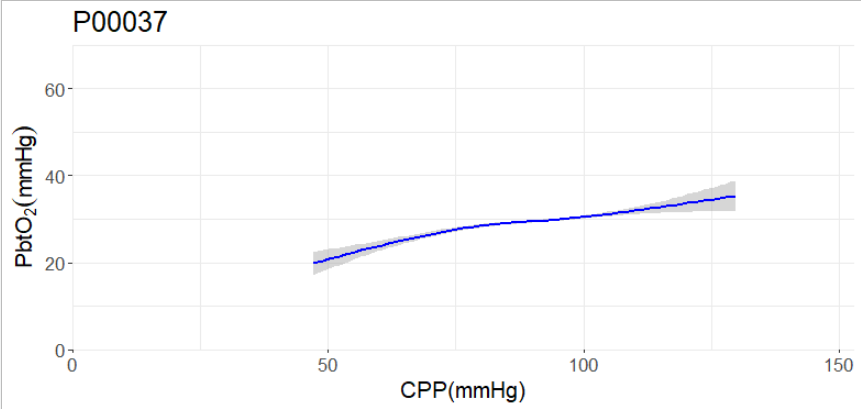


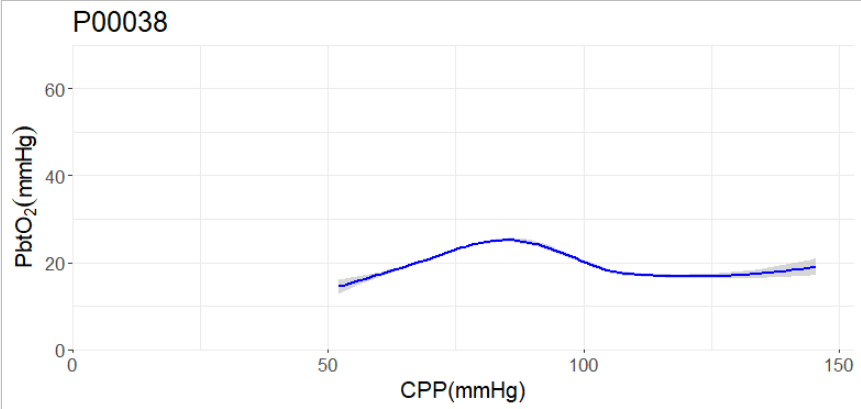


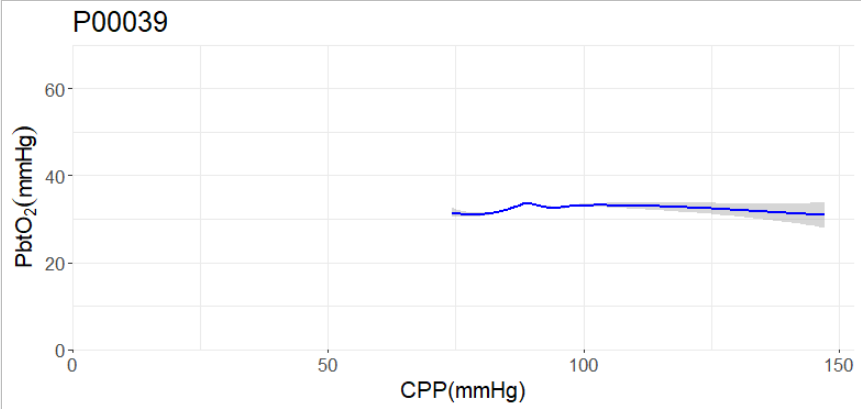


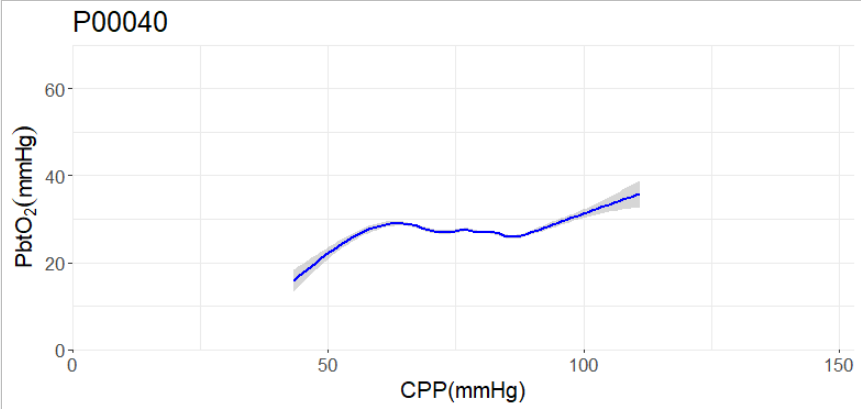


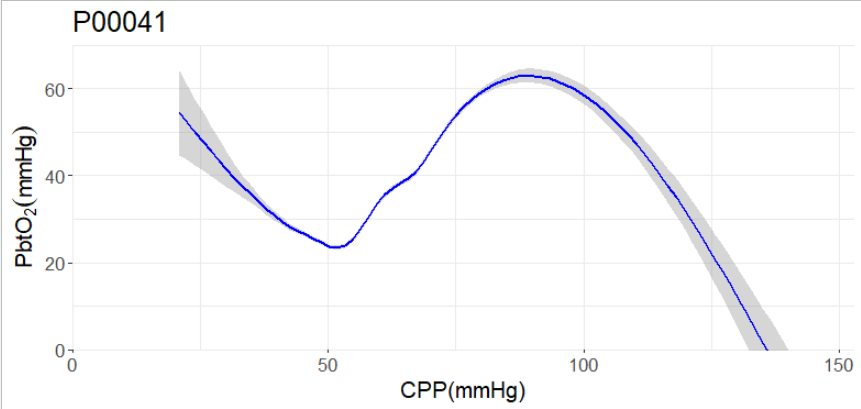


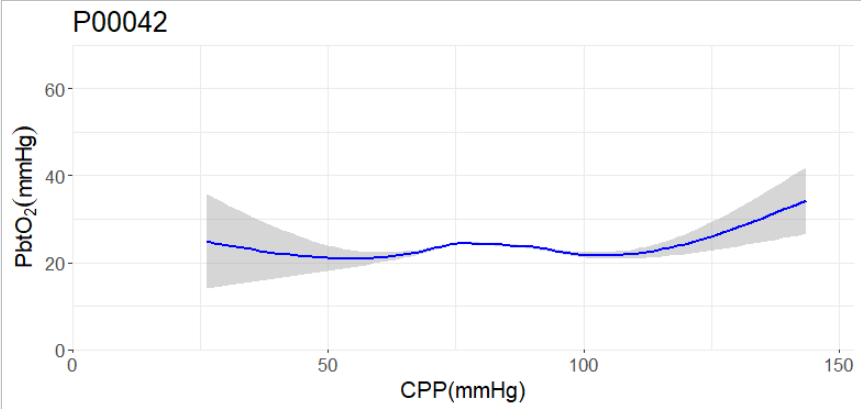


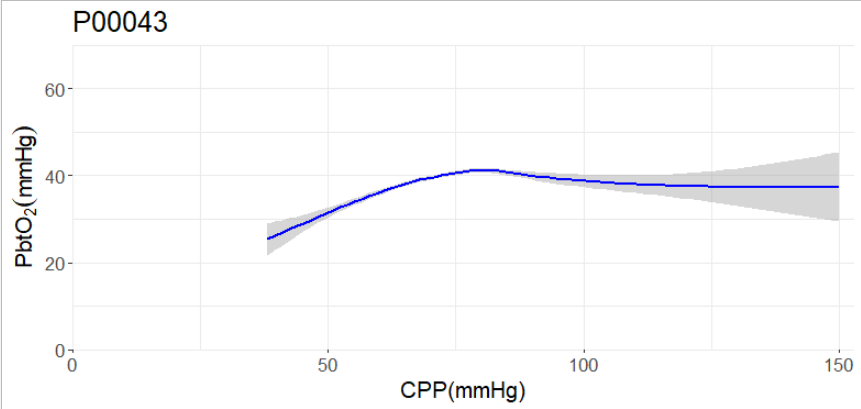


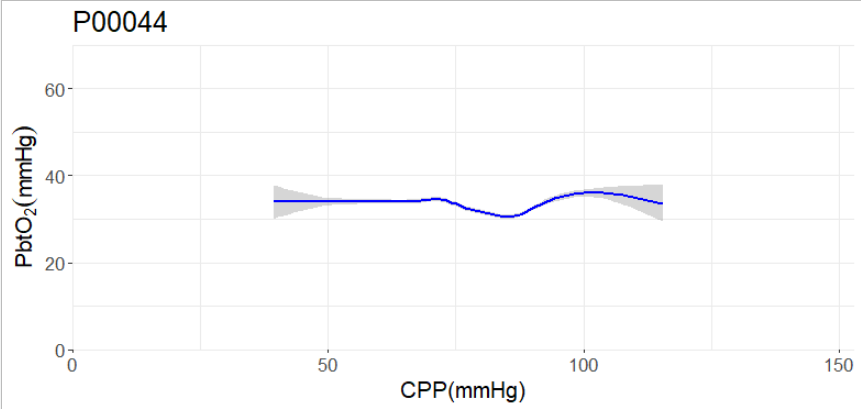


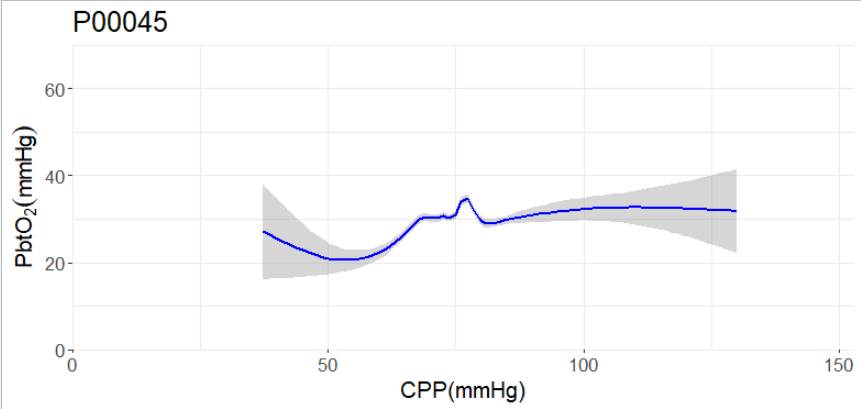


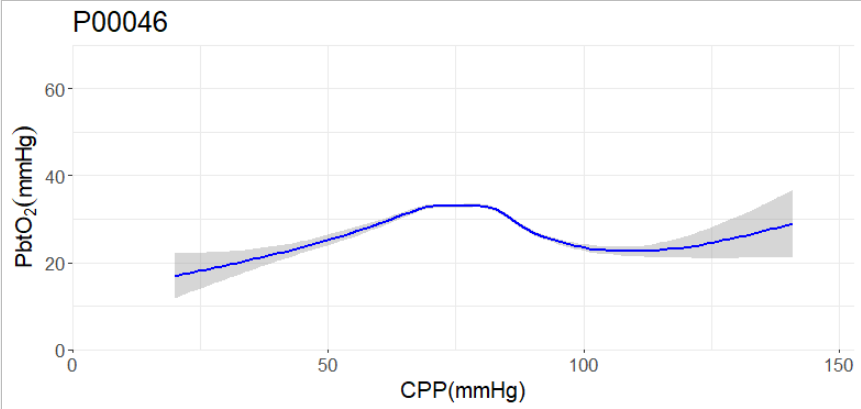


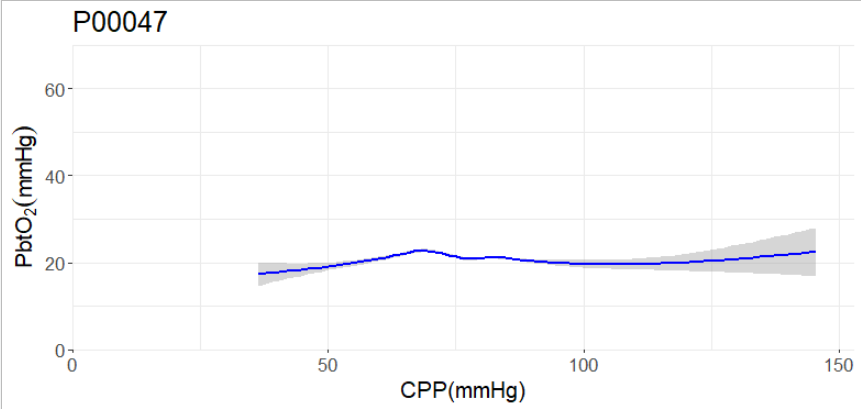


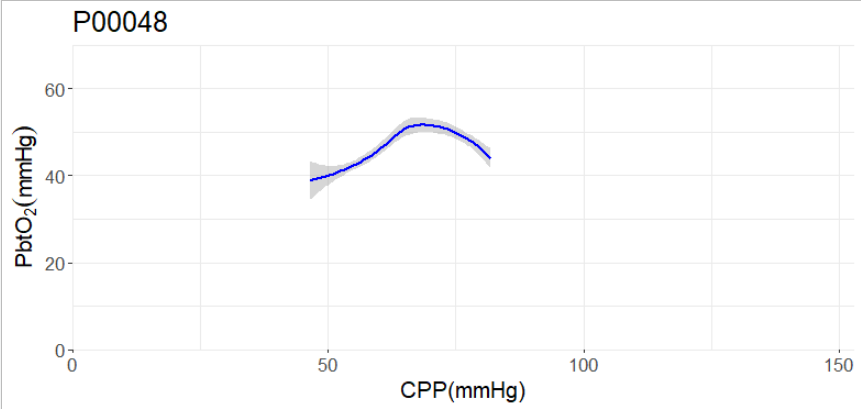


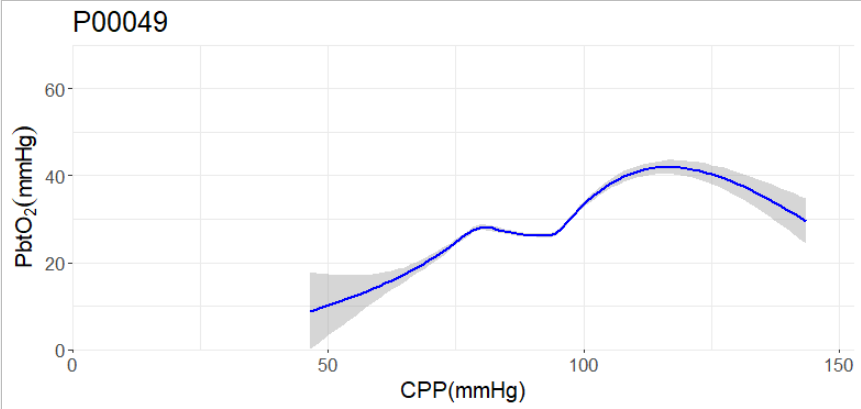


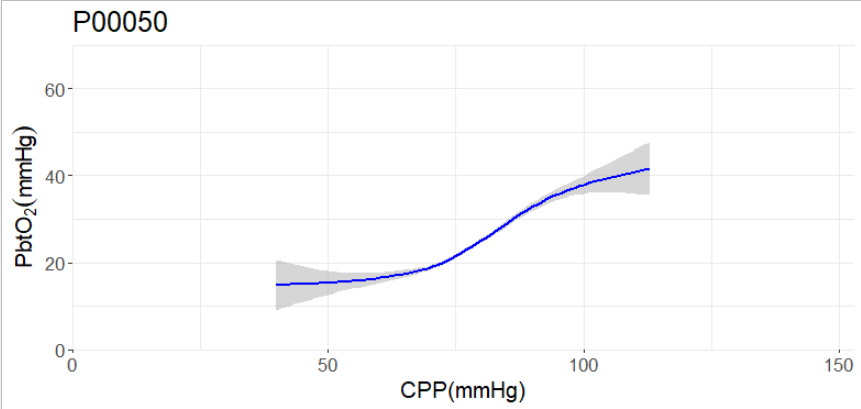


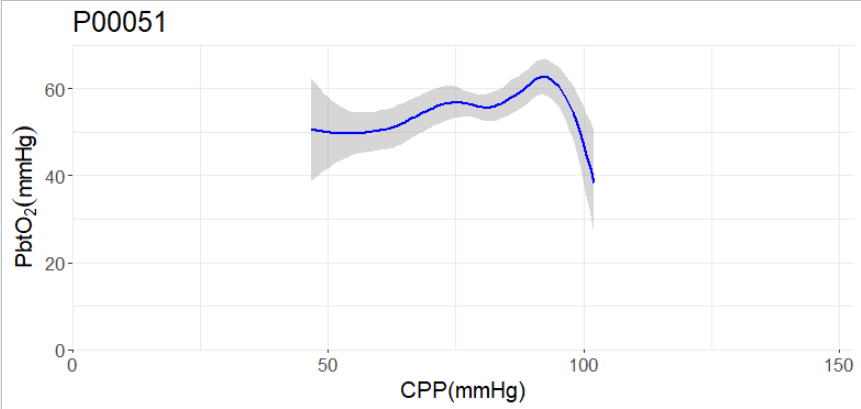


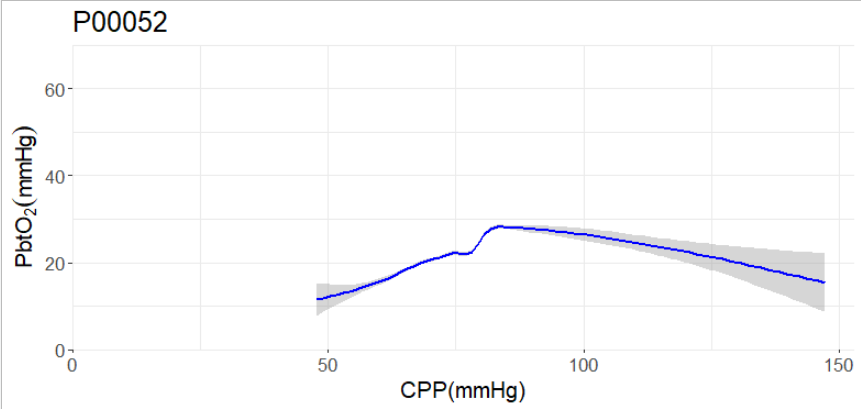


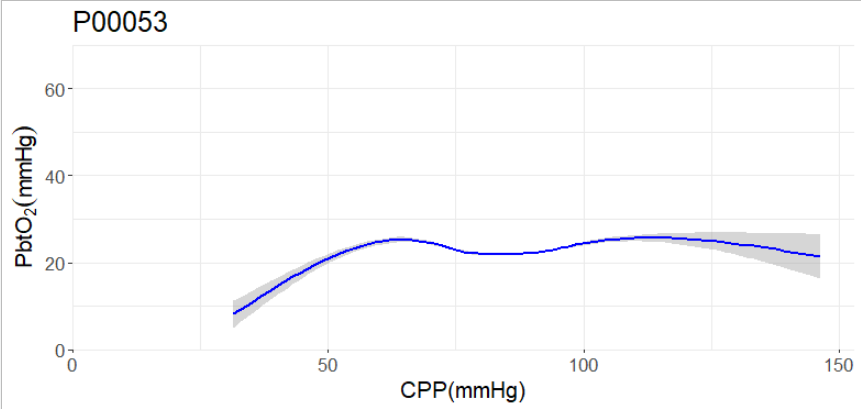


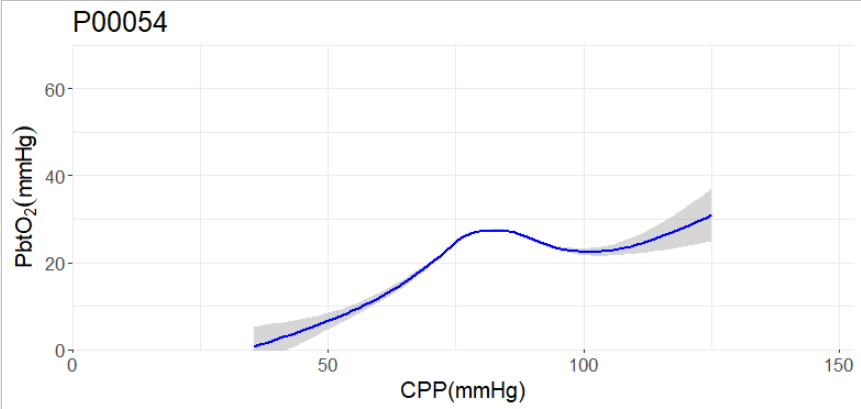


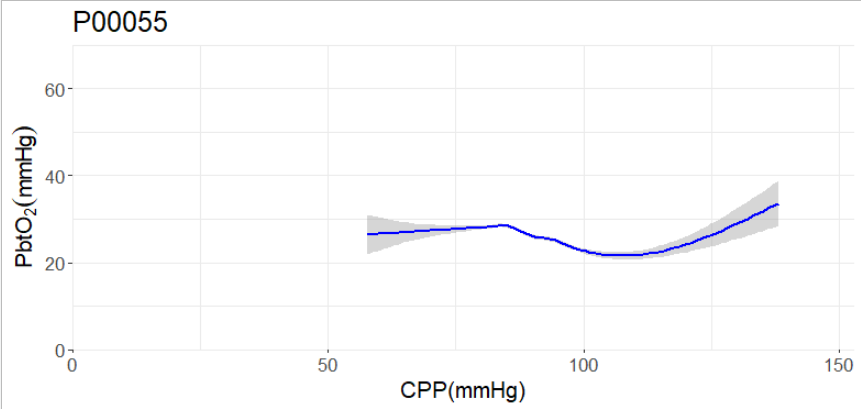


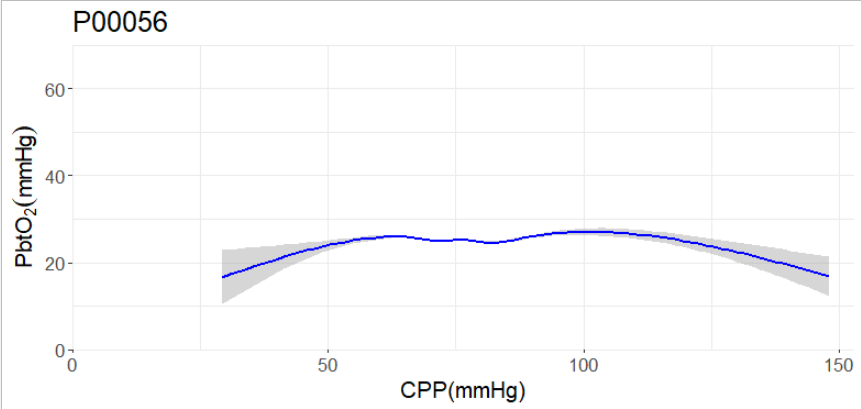


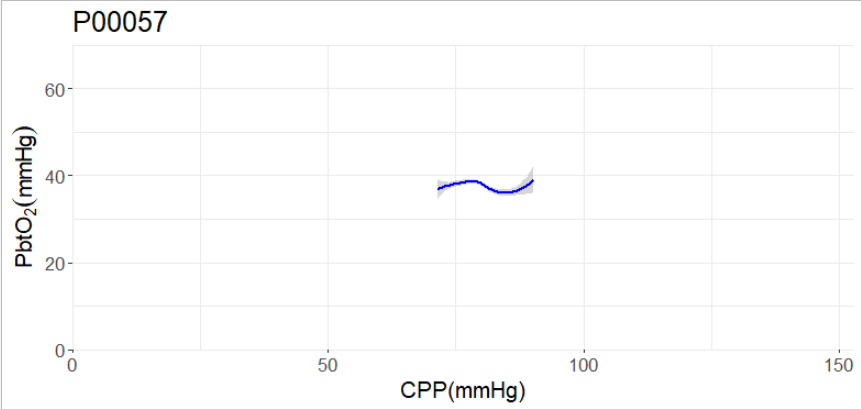


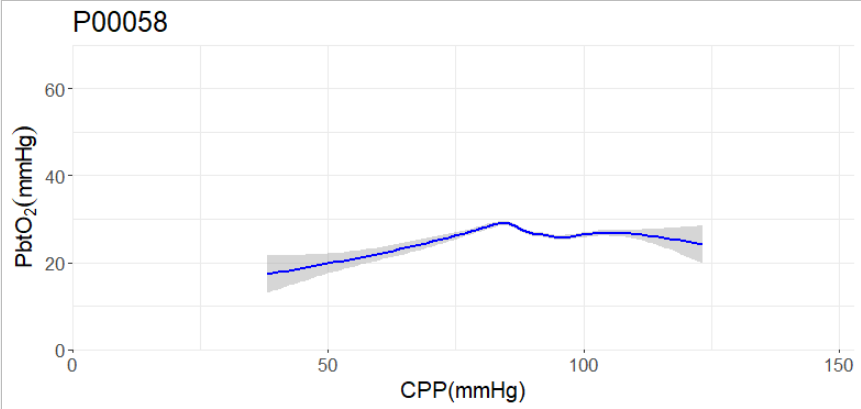


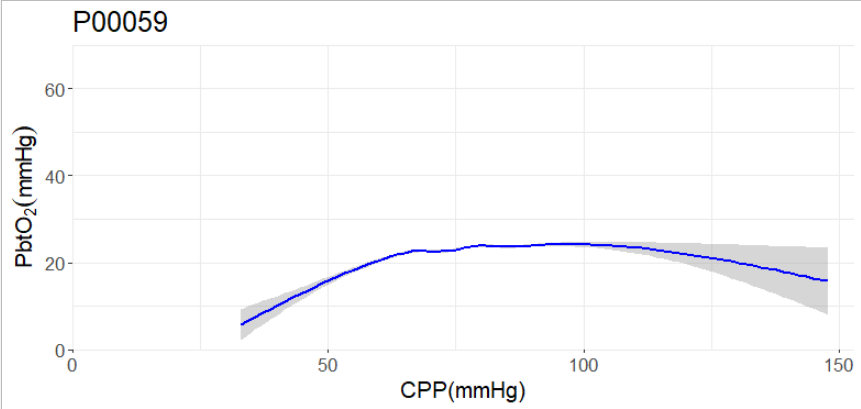


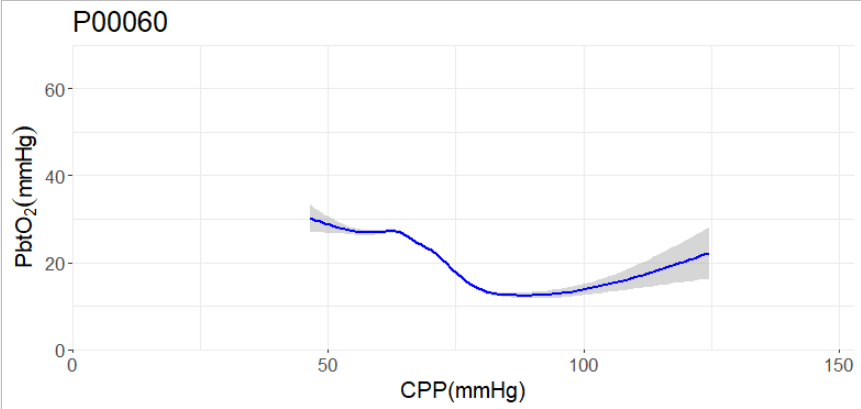


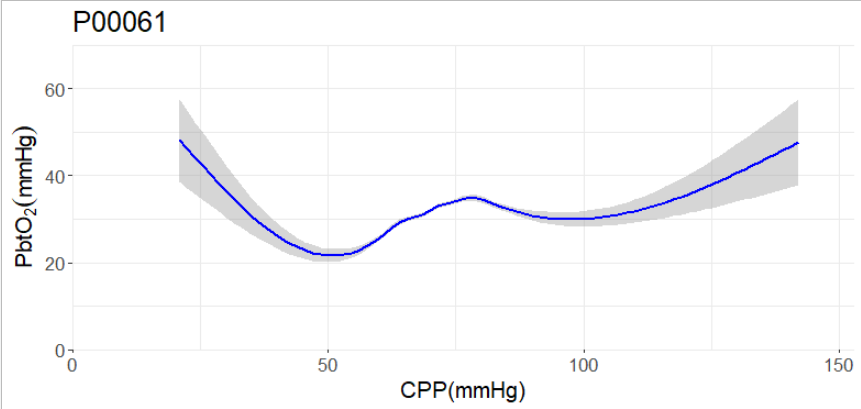


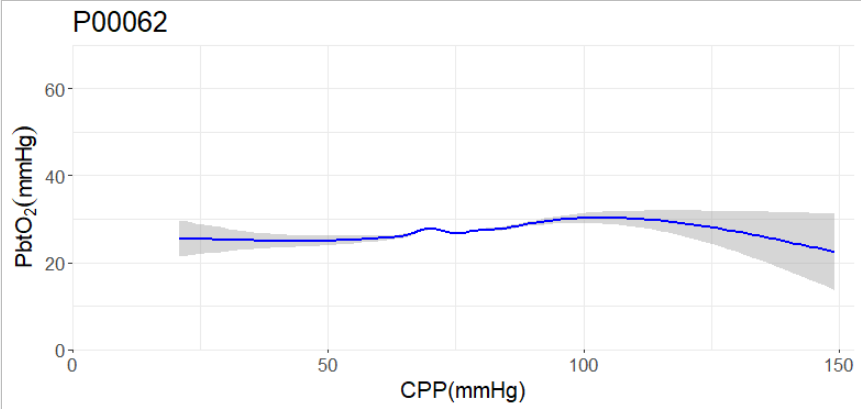


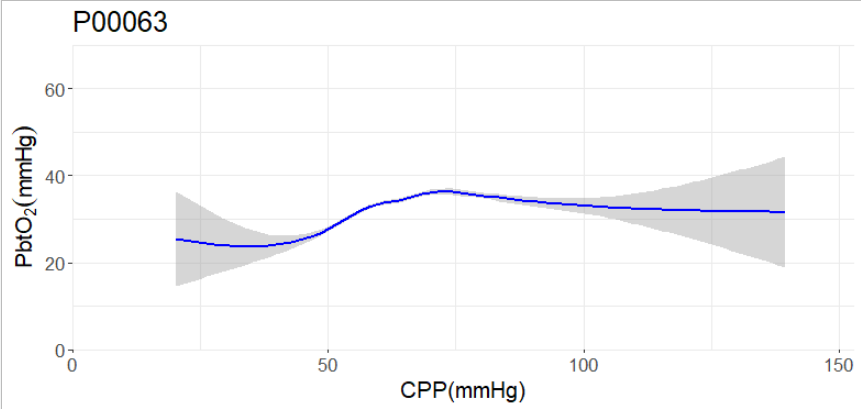


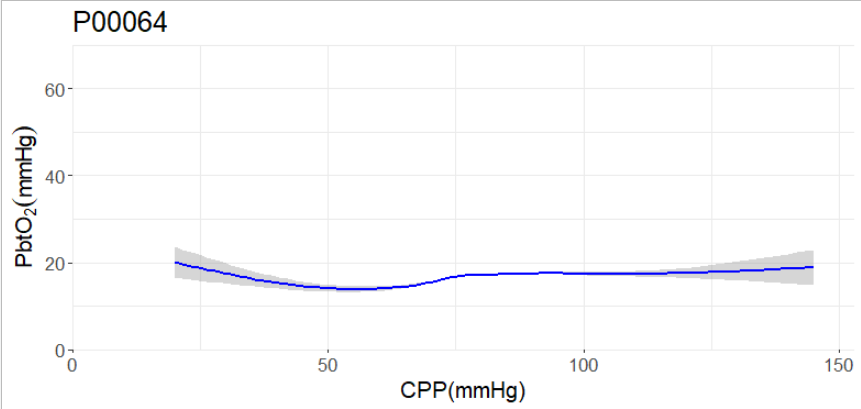


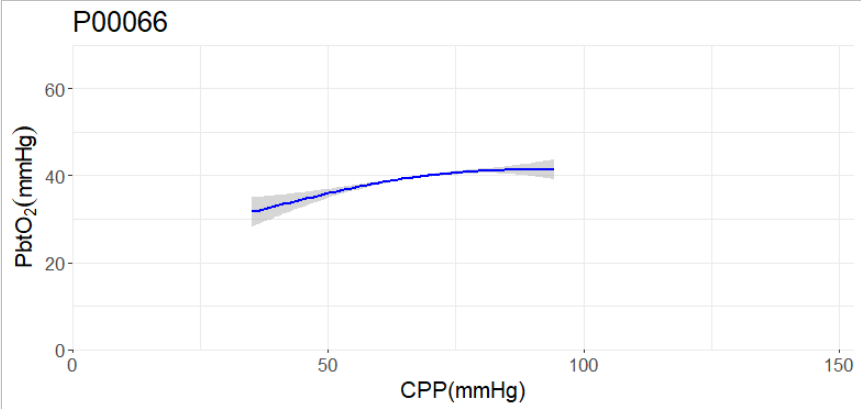


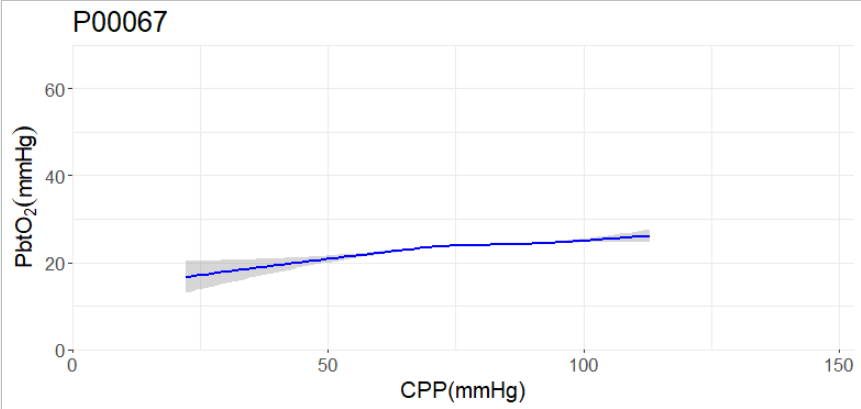


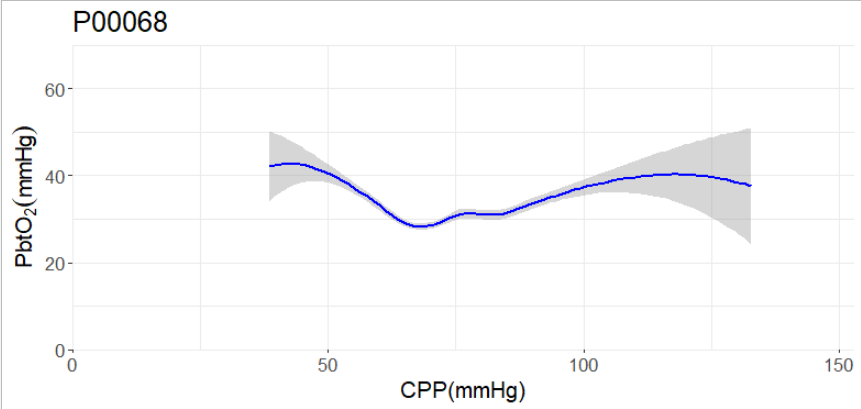


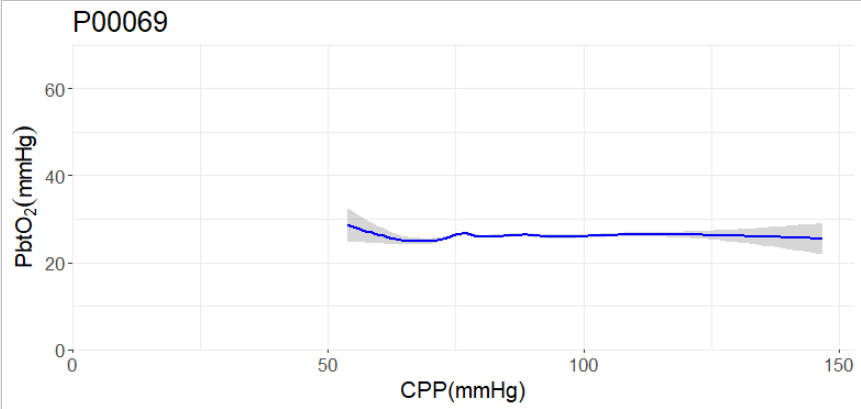


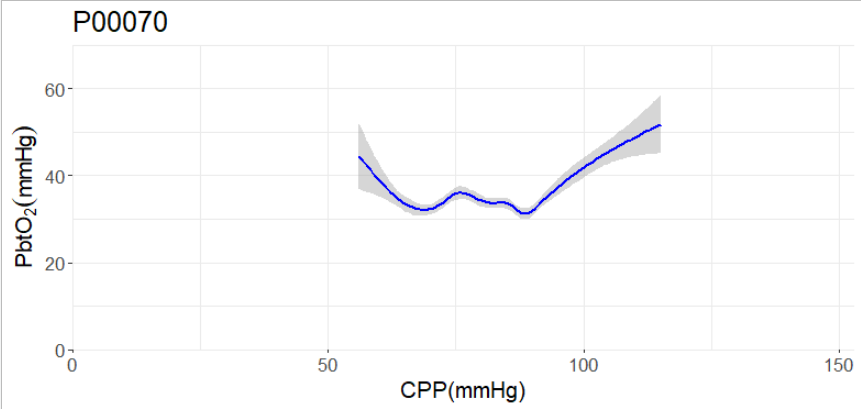


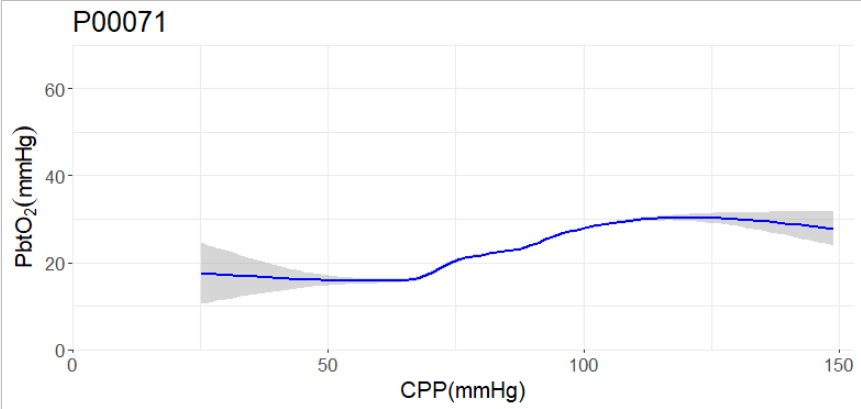


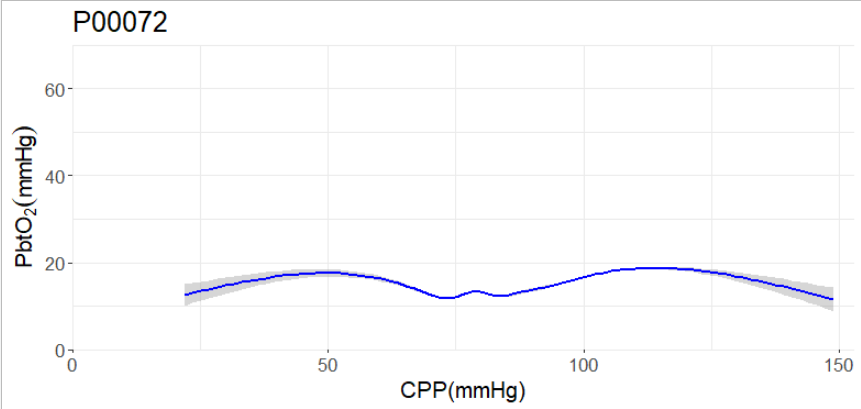


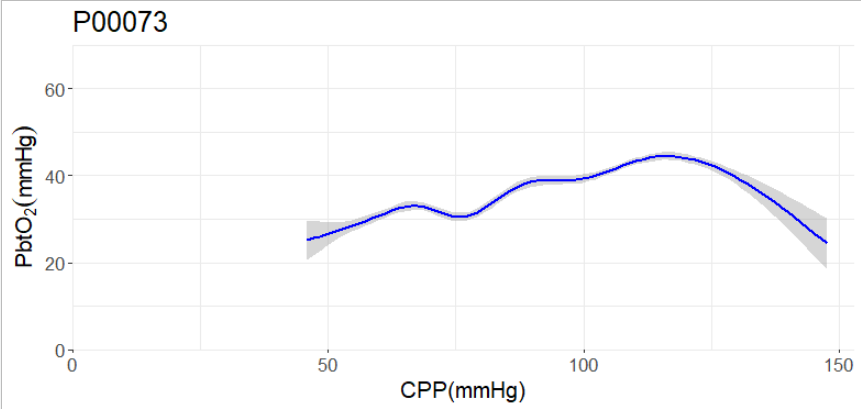


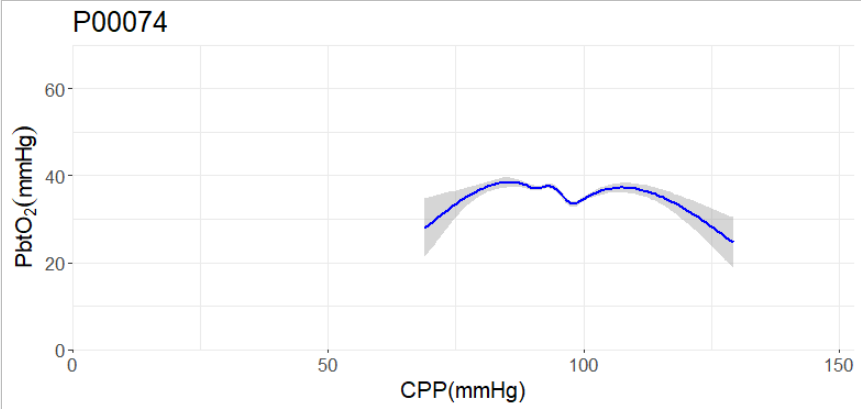


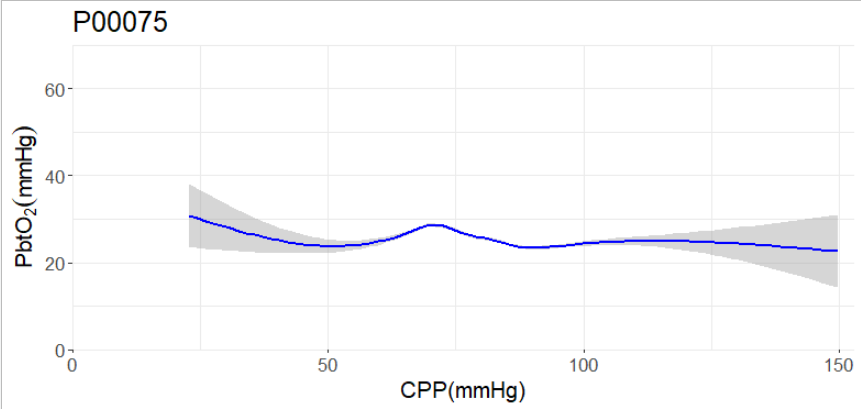


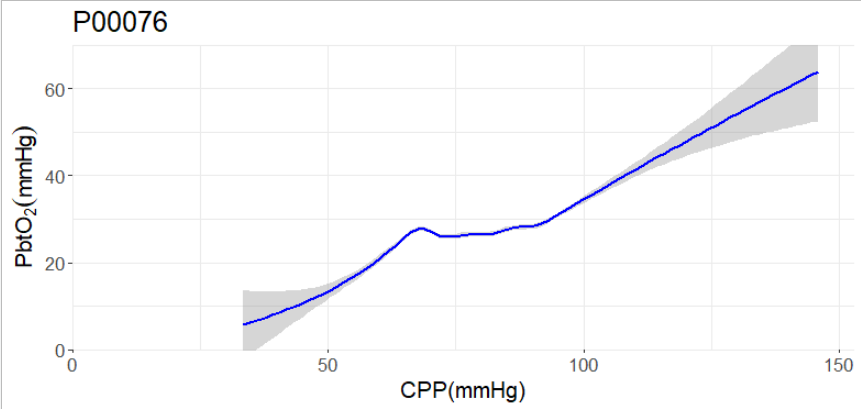


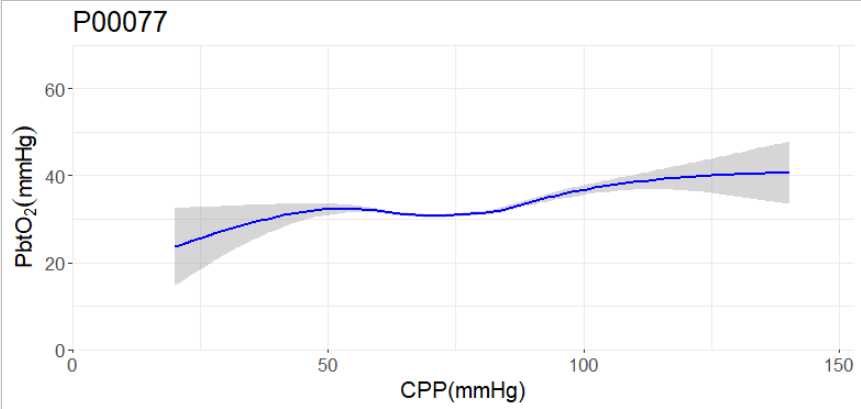


**Appendix B – Plots of PbtO_2_ versus ΔCPPopt for individual patients**


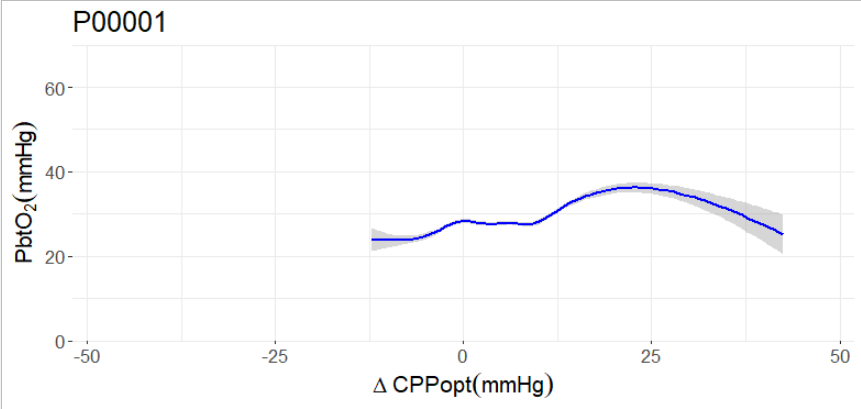


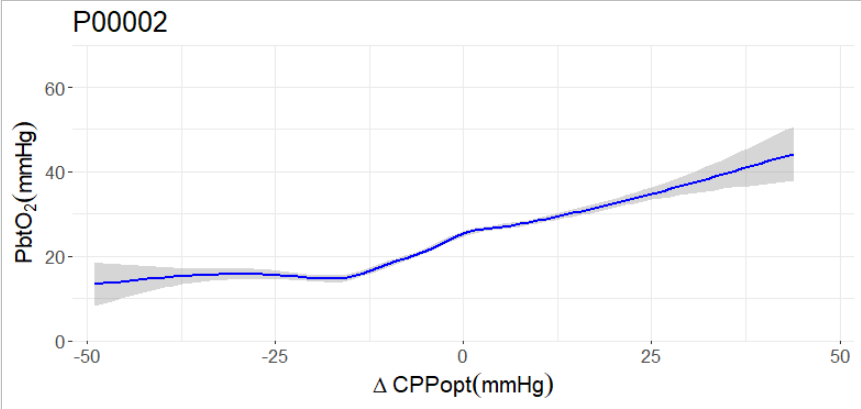


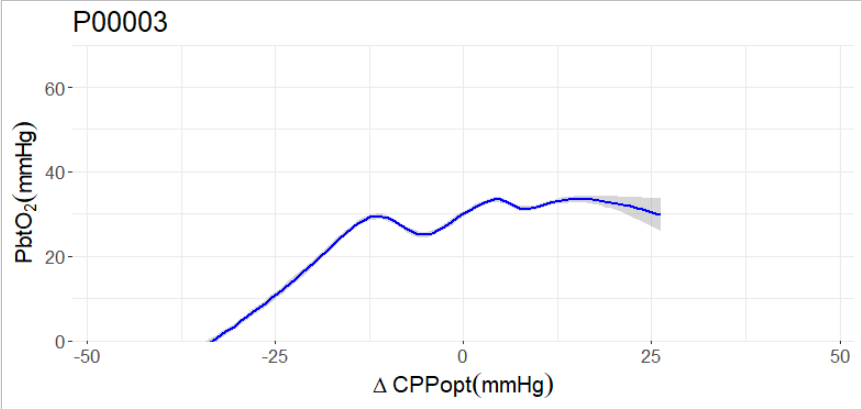


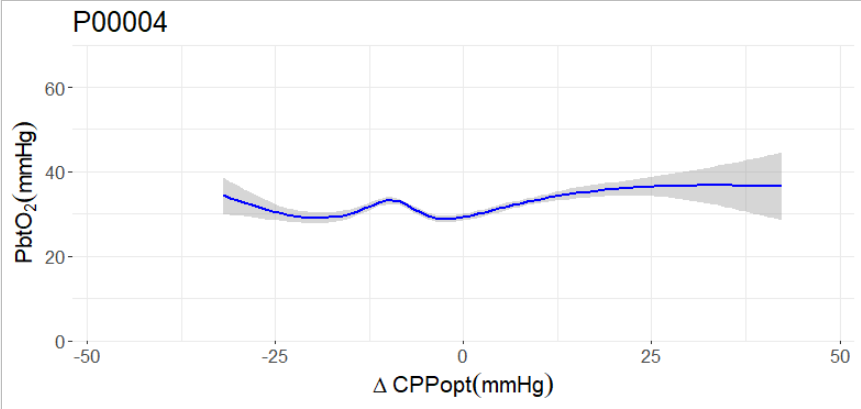


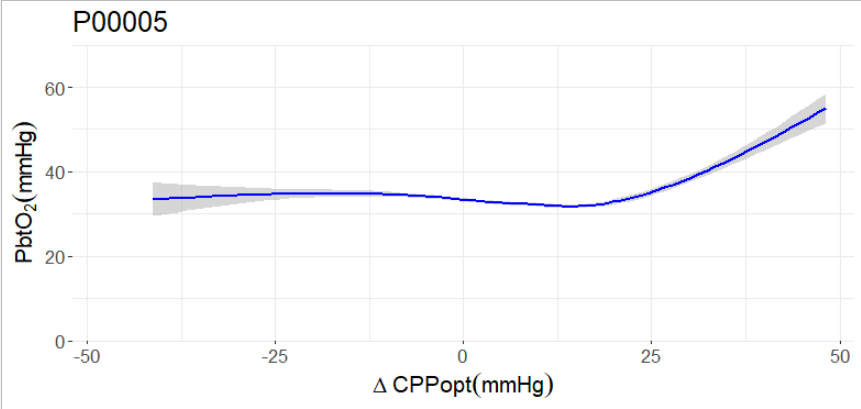


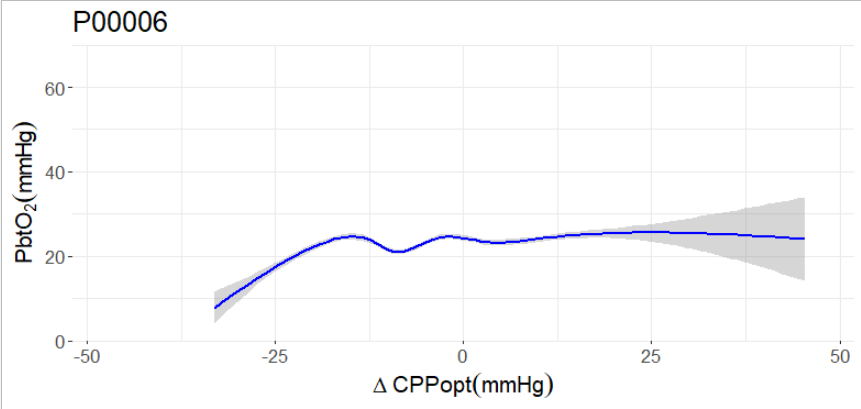


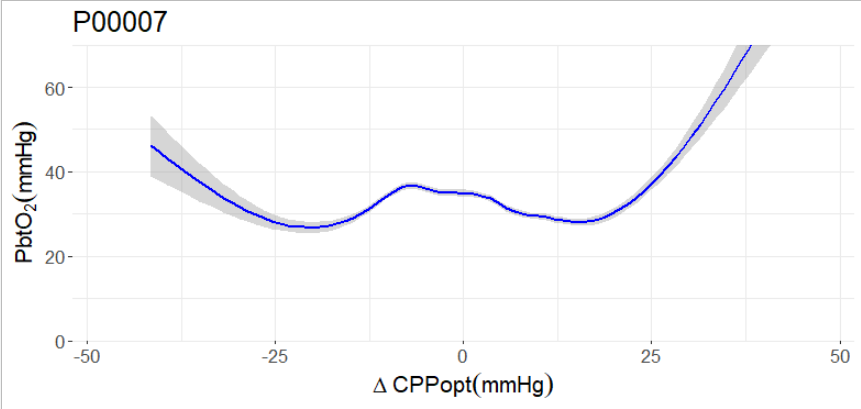


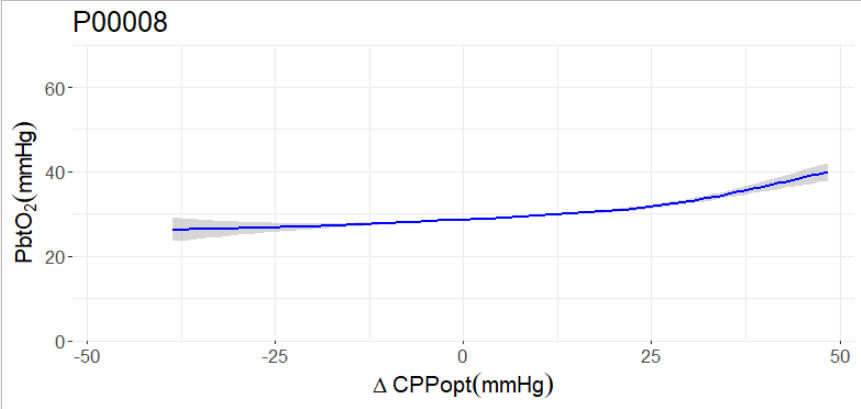


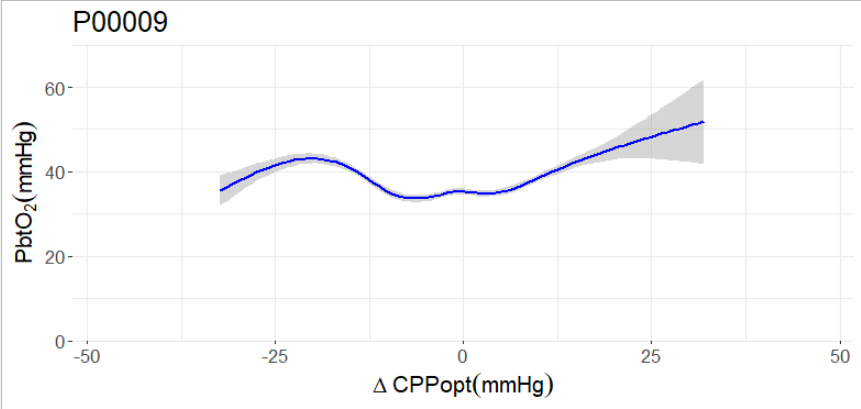


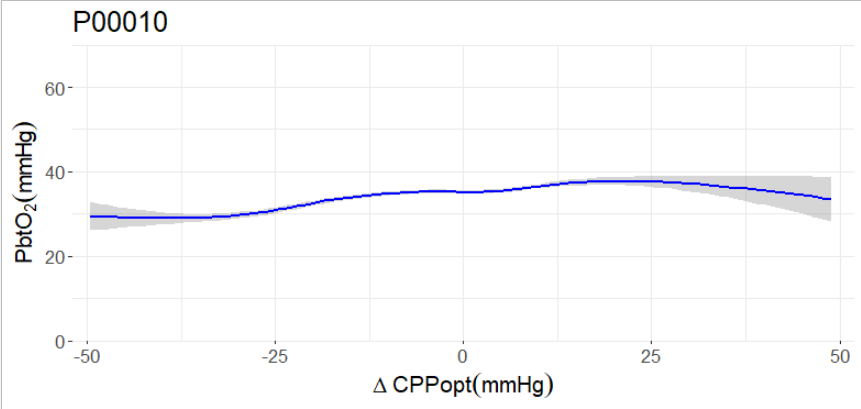


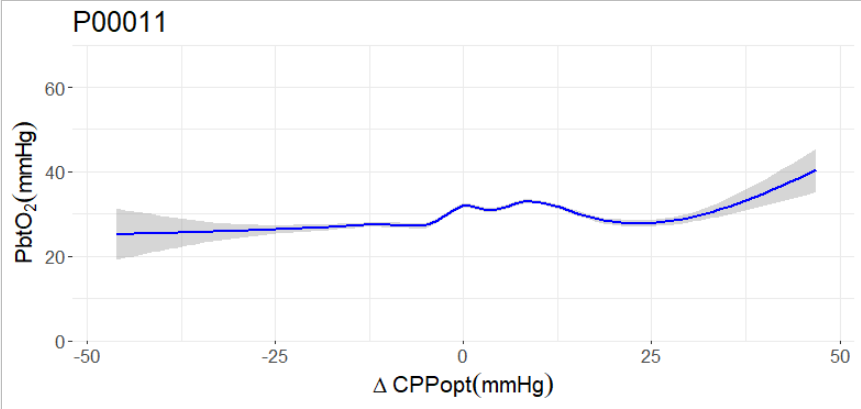


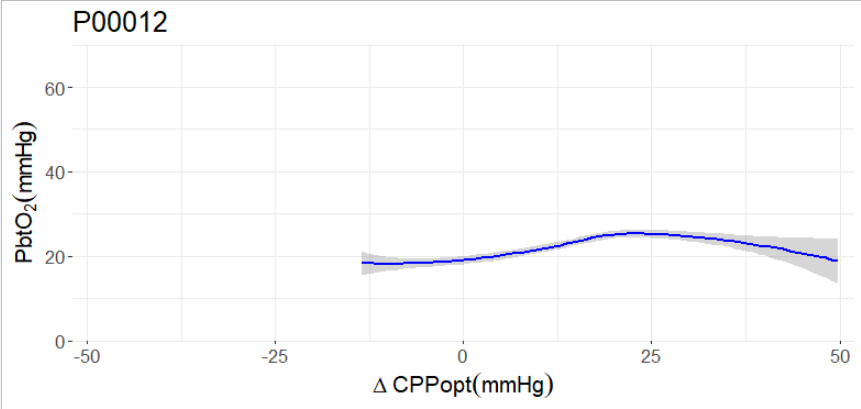


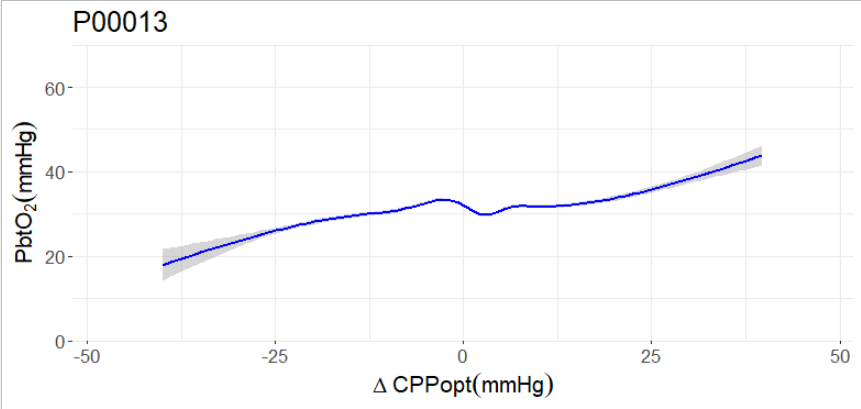


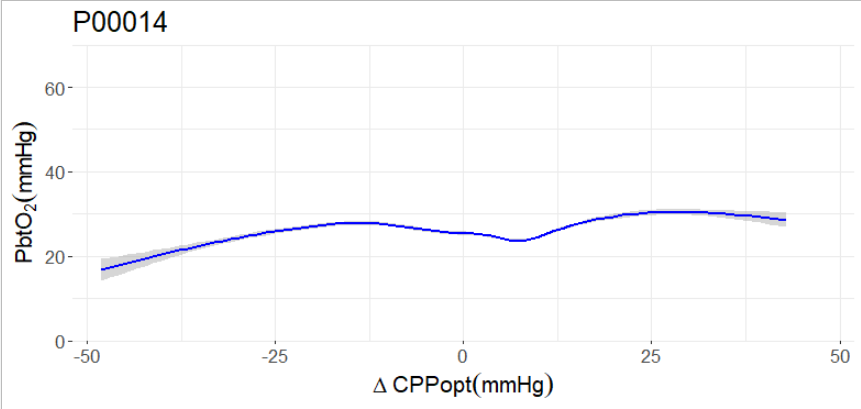


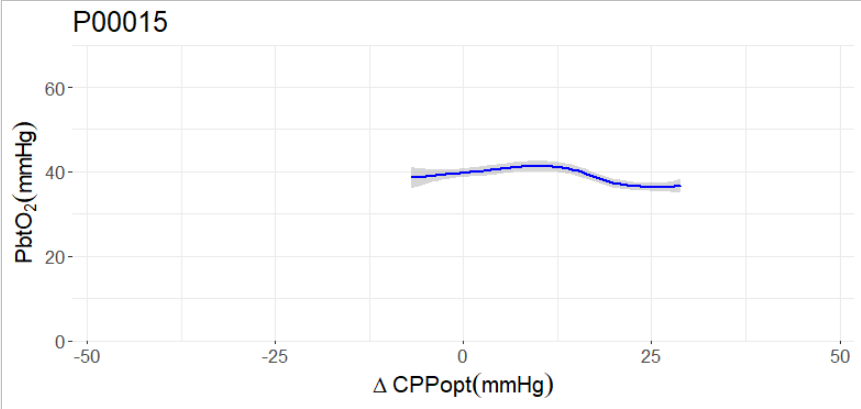


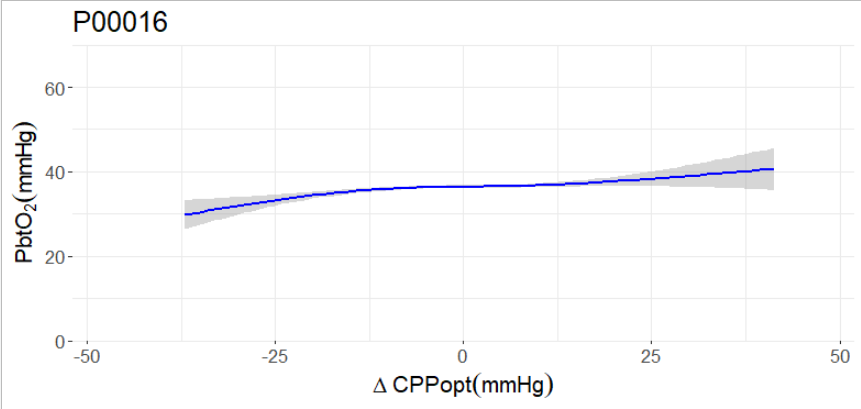


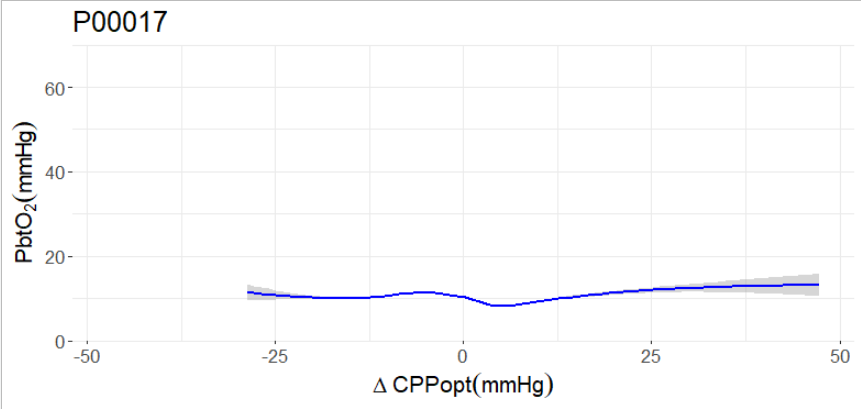


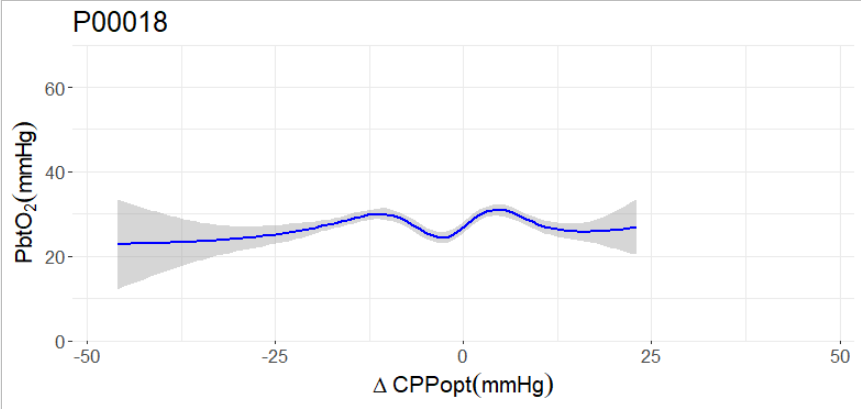


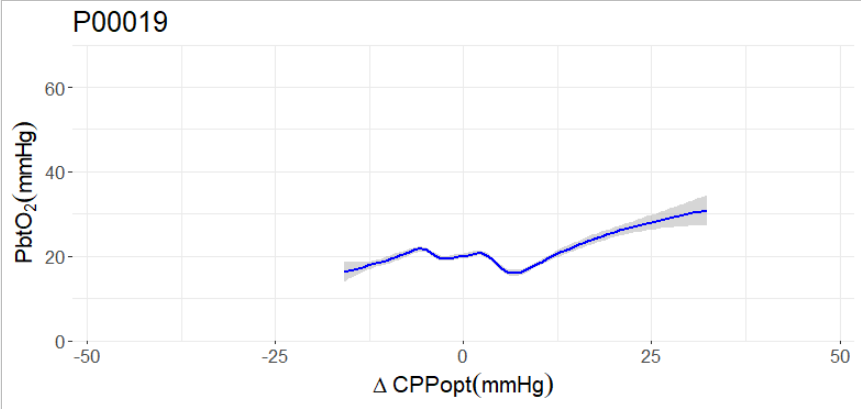


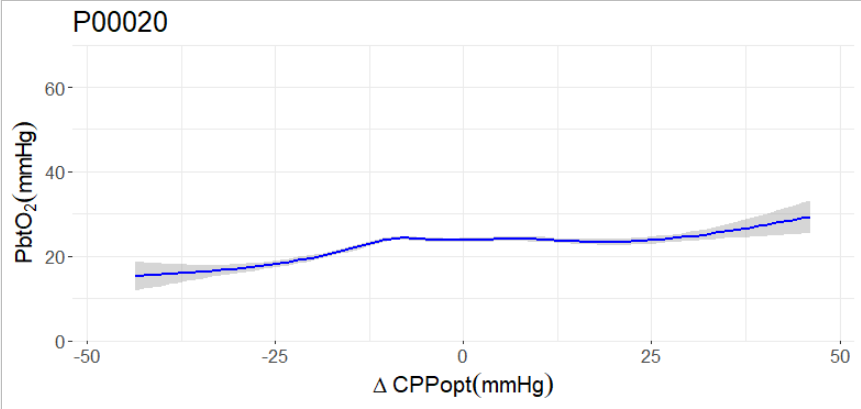


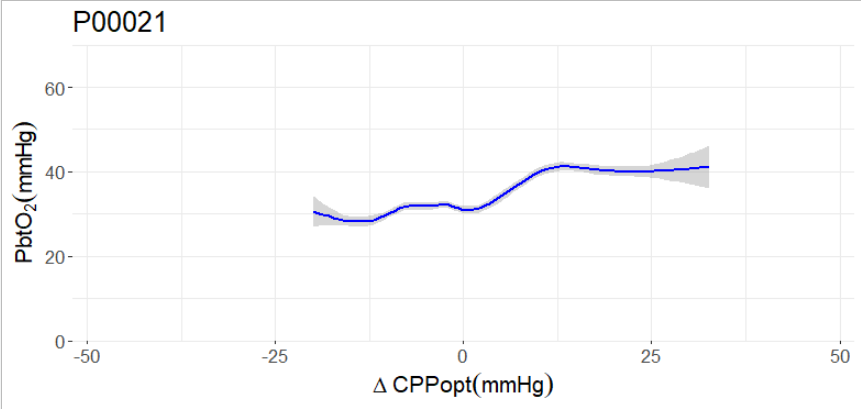


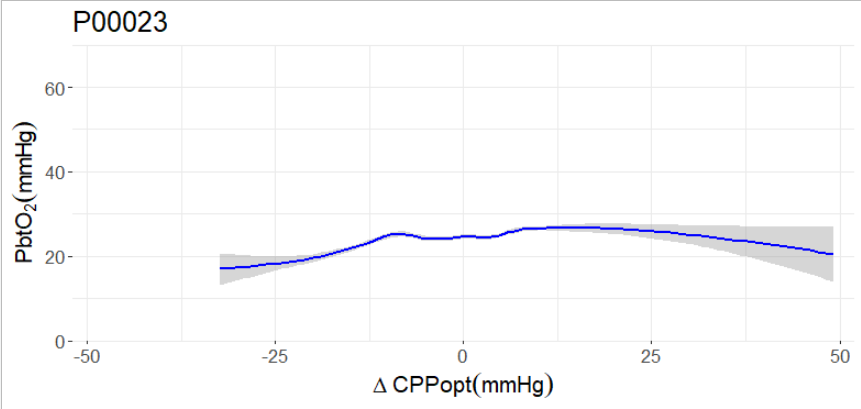


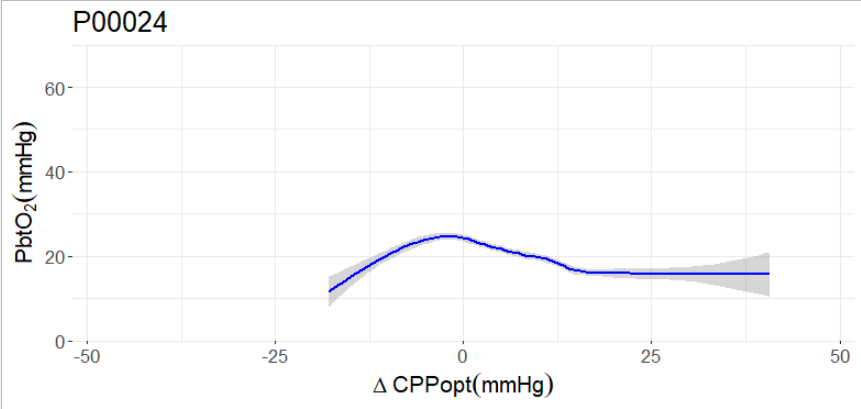


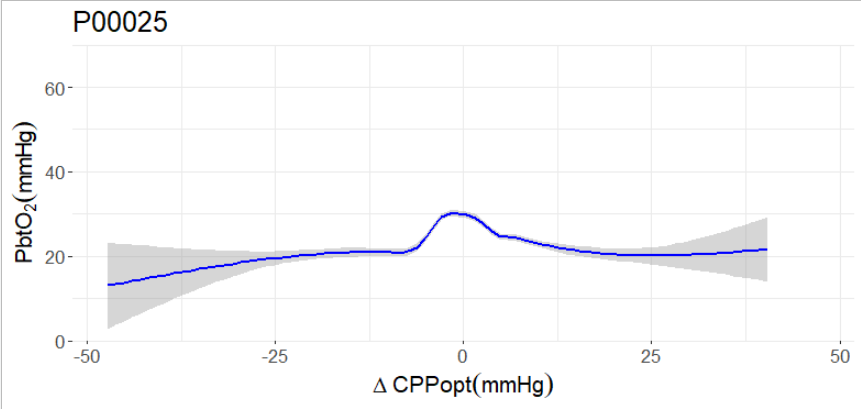

Supplement: Supplementary file 1 — Additional file 1: Appendix SA. Plots of PbtO2 versus CPP for individual patients. Appendix SB. Plots of PbtO2 versus ΔCPPopt for individual patients. [file 40635_2022_482_MOESM1_ESM.docx]
